# Coordinated Hibernation of Transcriptional and Translational Apparatus during Growth Transition of Escherichia coli to Stationary Phase

**DOI:** 10.1128/mSystems.00057-18

**Published:** 2018-09-11

**Authors:** Hideji Yoshida, Tomohiro Shimada, Akira Ishihama

**Affiliations:** a Department of Physics, Osaka Medical College, Takatsuki, Osaka, Japan; b Meiji University, School of Agriculture, Kawasaki, Kanagawa, Japan; c Research Center for Micro-Nano Technology, Hosei University, Koganei, Tokyo, Japan; Princeton University

**Keywords:** *Escherichia coli*, PS-TF screening, anti-sigma factor Rsd, ribosome modulation factor RMF, stationary-phase adaptation

## Abstract

In the process of Escherichia coli K-12 growth from exponential phase to stationary, marked alteration takes place in the pattern of overall genome expression through modulation of both parts of the transcriptional and translational apparatus. In transcription, the sigma subunit with promoter recognition properties is replaced from the growth-related factor RpoD by the stationary-phase-specific factor RpoS. The unused RpoD is stored by binding with the anti-sigma factor Rsd. In translation, the functional 70S ribosome is converted to inactive 100S dimers through binding with the ribosome modulation factor (RMF). Up to the present time, the regulatory mechanisms of expression of these two critical proteins, Rsd and RMF, have remained totally unsolved. In this study, attempts were made to identify the whole set of transcription factors involved in transcription regulation of the *rsd* and *rmf* genes using the newly developed promoter-specific transcription factor (PS-TF) screening system. In the first screening, 74 candidate TFs with binding activity to both of the *rsd* and *rmf* promoters were selected from a total of 194 purified TFs. After 6 cycles of screening, we selected 5 stress response TFs, ArcA, McbR, RcdA, SdiA, and SlyA, for detailed analysis *in vitro* and *in vivo* of their regulatory roles. Results indicated that both *rsd* and *rmf* promoters are repressed by ArcA and activated by McbR, RcdA, SdiA, and SlyA. We propose the involvement of a number of TFs in simultaneous and coordinated regulation of the transcriptional and translational apparatus. By using genomic SELEX (gSELEX) screening, each of the five TFs was found to regulate not only the *rsd* and *rmf* genes but also a variety of genes for growth and survival.

**IMPORTANCE** During the growth transition of E. coli from exponential phase to stationary, the genome expression pattern is altered markedly. For this alteration, the transcription apparatus is altered by binding of anti-sigma factor Rsd to the RpoD sigma factor for sigma factor replacement, while the translation machinery is modulated by binding of RMF to 70S ribosome to form inactive ribosome dimer. Using the PS-TF screening system, a number of TFs were found to bind to both the *rsd* and *rmf* promoters, of which the regulatory roles of 5 representative TFs (one repressor ArcA and the four activators McbR, RcdA, SdiA, and SlyA) were analyzed in detail. The results altogether indicated the involvement of a common set of TFs, each sensing a specific environmental condition, in coordinated hibernation of the transcriptional and translational apparatus for adaptation and survival under stress conditions.

## Abstract (precis)

During the growth transition of E. coli from exponential phase to stationary, the genome expression pattern is altered markedly. For this alteration, the transcription apparatus is altered by binding of anti-sigma factor Rsd to the RpoD sigma factor for sigma factor replacement, while the translation machinery is modulated by binding of RMF to 70S ribosome to form inactive ribosome dimer. Using the PS-TF screening system, a number of TFs were found to bind to both the *rsd* and *rmf* promoters, of which the regulatory roles of 5 representative TFs (one repressor ArcA and the four activators McbR, RcdA, SdiA, and SlyA) were analyzed in detail. The results altogether indicated the involvement of a common set of TFs, each sensing a specific environmental condition, in coordinated hibernation of the transcriptional and translational apparatus for adaptation and survival under stress conditions.

## INTRODUCTION

Bacteria maintain a sophisticated genetic system to optimize the rate of cell growth in response to environmental conditions. The growth rate is closely related to the intracellular level of the apparatus for gene expression. The early studies revealed the tight correlation between the level of ribosomes, the key apparatus of translation, and the rate of cell growth in a growing bacterial cell so as to satisfy the demand for protein synthesis under a given environmental condition (1, 2). The fast-growing Escherichia coli K-12 strain contains as many as 70,000 ribosomes per cell, while at lower growth rates, this number is reduced to less than 20,000 (3, 4). Likewise the intracellular level of RNA polymerase (RNAP) core enzyme, the key apparatus of transcription, is maintained through autogenous regulation at a level of 2,000 molecules per genome (5, 6), which correlates with the rate of cell growth (7). The intracellular levels of both transcription apparatus and translational machinery, however, change in coordinate fashion in response to variation in cell growth rate and in coupling with growth phase transition from the exponential phase to stationary phase. One of the most common stresses leading to entry into the stationary phase is limited availability of nutrients (8). In exponentially growing E. coli K-12 cells, the growth-related genes are highly expressed, which are, however, turned off upon entry to the stationary-phase cells; instead, a set of stationary-phase-specific genes are expressed (5, 9). The growth-related alteration of genome expression takes place through modulation of the level of functional forms of the transcriptional and translational apparatus and modulation of utilization of the remaining transcriptional and translational apparatus. As to control of the level, two modes of the regulation are involved: the shutdown of further production of the transcriptional and translational apparatus and the conversion of unused excess apparatus into nonfunctional conformations for storage.

The RNAP holoenzyme is composed of the core enzyme with the catalytic activity of RNA synthesis and one of seven species of the sigma subunit with the activity of promoter recognition (10, 11). Replacement of the RNAP-associated sigma subunit is the most efficient way for alteration of the promoter recognition property of RNAP. The stationary phase is achieved through replacement of RNAP-associated sigma factor from RpoD, the major sigma factor for recognition of growth-related genes, to the stationary-phase-specific RpoS (7, 12, 13). In the process of sigma factor replacement, we identified the involvement of an anti-sigma factor, designated Rsd (regulator of sigma D), for conversion of unused RpoD to an inactive form for storage (14). The function of Rsd is to sequester the RpoD sigma factor and displace RNAP core enzyme, which in turn becomes accessible for the stationary-phase-specific RpoS sigma factor (14, 15). The promoter selectivity of RNAP holoenzyme is further modified after interaction with a total of approximately 300 species of transcription factors (TFs) (10, 11). Different sets of TFs are involved in regulation of the growth-related genes and stationary-phase-specific genes.

At the translation level, the adaptation to stationary phase is accompanied by the conversion of ribosomes into inactive forms. The functional form of ribosomes in growing E. coli K-12 cells is the 70S monomer, consisting of 30S and 50S subparticles, but upon entry into the stationary phase, the 70S ribosomes are converted into functionally inactive 100S dimers through the association of a small basic protein, RMF (ribosome modulation factor), 55 amino acid residues in length (16, 17). RMF binds near the ribosomal proteins S13, L13, and L2, close to the peptidyl-tRNA binding site (18). Since a mutant E. coli strain that is defective in the *rmf* gene is unable to survive in the stationary phase, the dimerization of ribosomes is essential for stationary-phase survival of E. coli (19). Taking these observations together, we proposed that Rsd and RMF play key roles in storage of the unused apparatus of transcription and translation in inactive forms (10, 11, 20, 21).

Up to the present time, however, little has been known about the regulation of expression of Rsd and RMF. Both the *rsd* and *rmf* genes form single-gene transcription units. Expression of RMF is positively regulated by the stringent response alarmone (p)ppGpp (guanosine-3′,5′-bisdiphosphate or guanosine pentaphosphate) (22) and by the carbon source-sensing cAMP-cAMP receptor protein complex (cAMP-CRP) (23). The signal for stringent control, ppGpp, is synthesized upon exposure to a defect in nutrients, in particular amino acids, and plays a key role for immediate shutdown of further synthesis of the gene expression apparatus (24–26). ppGpp directly binds to either RpoZ (omega) subunit of RNAP (27) or RNAP-associated small regulatory protein DksA (28) and modulates the promoter selectivity so as not to transcribe the genes for RNAP and ribosomes (29). On the other hand, CRP is activated by cAMP that is synthesized upon exposure to defect in favorable carbon sources such as glucose (30). Based on a systematic search of regulatory targets for more than 200 TFs from E. coli K-12 strain W3110, we have proposed that most of the E. coli promoters are under the control of multiple species of TF, each monitoring an environmental factor or condition (31, 32). Since the synthesis of both Rsd and RMF should be controlled in response to a variety of environmental factors and conditions during the transition from exponential growth to stationary phase, we predicted the involvement of a number of TFs in the regulation of expression of the *rsd* and *rmf* genes.

For quick shortcut screening of regulatory proteins for each promoter in E. coli K-12, we developed two *in vitro* systems: the “genomic SELEX” (currently designated gSELEX) system for searching of regulatory target promoters by a test sigma factor or TF and the promoter-specific transcription factor (PS-TF) screening system for searching of TFs involved in regulation of a test promoter. By using the gSELEX screening *in vitro*, we have identified the whole set of regulatory targets for RNAP sigma factors (33, 34) and more than 200 TFs (32 [also see the TEC database at www.shigen.nig.ac.jp/ecoli/tec/]). In parallel with the gSELEX screening, we developed the PS-TF screening system for searching of TFs involved in regulation of one specific promoter (35). The successful development of these two experimental systems for genome-scale analysis of the TF network relied on the established concept that DNA-binding TFs in E. coli generally bind near promoters for effective interplay with promoter-bound RNAP (36, 37). Here we employed this PS-TF system as a shortcut approach for identification of the whole set of TFs involved in regulation of the *rsd* and *rmf* promoters. After screening of 194 TFs, we identified a total of as many as 74 possible candidate TFs that regulate both the *rsd* and *rmf* promoters. For detailed analysis of the simultaneous regulation of expression of anti-sigma factor Rsd and ribosome dimerization factor RMF, we focused on five stress response TFs, each of which is involved in regulation of a different set of stress response genes for adaptation: ArcA (*a*erobic *r*espiratory *c*ontrol), McbR (*M*qsR-controlled *c*olonic acid and *b*iofilm *r*egulator), RcdA (*r*egulator of *c*sg*D*), SdiA (*s*uppressor of cell *di*vision inhibitor), and SlyA (Salmonella hemo*ly*tic protein). Regulatory functions have been analyzed in detail for these TFs.

## RESULTS

### Screening of TFs involved in regulation of the *rsd* and *rmf* promoters.

**FIG 1:**
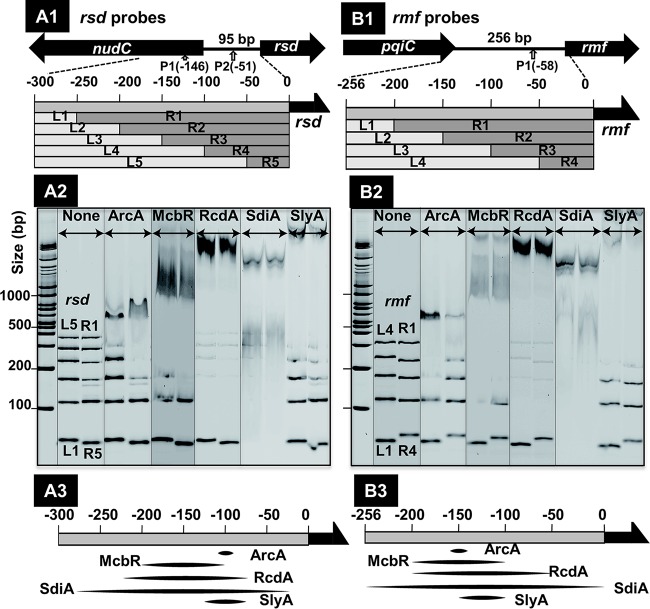
Mapping of TF-binding sites on the *rsd* and *rmf* promoters. (A1 and B1) Location of the probes used for mapping of the binding sites of 5 TFs (ArcA, McbR, RcdA, SdiA, and SlyA). The full-size probe of the *rsd* promoter (A1) corresponds to 300-bp-long sequence upstream from the initiation codon of the *rsd* gene, while the full-size probe of the *rmf* promoter (B1) corresponds to the 256-bp-long spacer sequence between the *pqiC* and *rmf* genes. The location of the RpoD promoter is shown by an upward arrow with the distance (in parentheses) from the translation initiation site. The full-size *rsd* probe was further divided into 5 segments, while the *rmf* probe was divided into 4 segments. In each segment, 5′-proximal and 3′-proximal segments were designated L and R, respectively. (A2 and B2). Using all of these probes, gel shift assays were performed for mapping of the binding sites of 5 TFs on the *rsd* (A2) and *rmf* (B2) probes. A mixture of 0.5 pmol each of FITC-labeled probes was incubated with 20 pmol of each TF and directly subjected to PAGE analysis. (A3 and B3) The binding regions of TFs on the *rsd* (A3) and *rmf* (B3) promoters were elicited from the results of gel shift assays.

**FIG 2:**
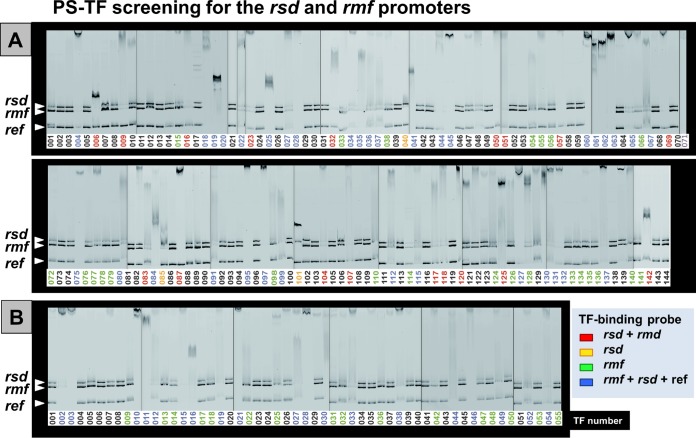
PS-TF screening of TFs with binding activity to the *rsd* and *rmf* promoters. Three FITC-labeled DNA probes (0.5 pmol each of 300-bp-long *rsd* promoter, 256-bp-long *rmf* promoter, and 193-bp-long internal reference DNA) were mixed with 20 pmol each of 194 species of purified TFs (listed in Table S1) in 10 μl of DNA-binding buffer, and after incubation at 37°C for 20 min, the DNA-protein mixtures were directly subjected to PAGE for detection of DNA-protein complexes under the standard running conditions (38). TFs with binding activity to the *rsd* probe alone, the *rmf* probe alone, and both of the *rsd* and *rmf* probes are shown in green, orange, and red, respectively, while TFs that showed binding activity to not only the *rsd* and *rmf* probes but also the reference probe are shown in blue.

For identification of the whole set of TFs involved in regulation of the *rsd* and *rmf* promoters, we employed in this study the PS-TF screening system *in vitro* (38), using *rsd* and *rmf* promoter probes and a total of 194 purified TFs of E. coli K-12 W3110 (see Table S1 in the supplemental material for the list of TFs used in this study). For detection of TF-probe complexes by the PAGE system, we used three species of fluorescein isothiocyanate (FITC)-labeled DNA fragment: i.e., a 300-bp-long *rsd* promoter, a 256-bp-long *rmf* promoter, and a 193-bp-long internal reference probe corresponding to an open reading frame sequence of the *rtcA* gene encoding RNA 3′-terminal phosphate cyclase (for the probe design, see Fig. S1 in the supplemental material). The activator-binding sites are generally present between −180 and −30 bp upstream of most of the known promoter, while the repressor-binding sites are located between positions −10 and +60 relative to the transcription start sites (for instance, see reference 39 and also RegulonDB [http://regulondb.ccg.unam.mx/]). Within the 300-bp-long *rsd* probe, two RpoD-dependent transcription start sites exist at −146 and −51 bp upstream, respectively, of the translation initiation site (Fig. 1A, panel A1). Thus, 154- and 197-bp-long sequences exist upstream of the P1 and P2 promoters. On the other hand, a single RpoD promoter exists within the 256-bp-long *rmf* probe at position −58 upstream of its initiation codon (Fig. 1B, panel B1), and thus, a 198-bp-long sequence exists upstream from P1. Thus, both *rsd* and *rmf* probes contain spaces enough for binding of most, if not all, of the known activators and repressors. A mixture of three FITC-labeled probes (0.5 pmol each) was mixed with 20 pmol each of the purified TFs, and after incubation for 20 min at 37°C, the mixtures were directly subjected to the mixed PAGE. Figure 2 shows a representative pattern of this mixed-PAGE analysis.

10.1128/mSystems.00057-18.2 TABLE S1 TFs used for PS-TF screening. (A) Characterized TFs. (B) Uncharacterized TFs. Note that the list of uncharacterized TFs in part B was prepared 4 years ago when we started this project, but the regulatory functions have since been identified for 14 TFs. Since the numbering of TFs used in PS-TF screening, as shown in Fig. 2, was used in this table, these 14 TFs remained as included in part B. The latest version of the TF list in E. coli K-12 is described in reference 32 and has been deposited in the TEC database (www.shigen.nig.ac.jp/ecoli/tec/). Download TABLE S1, PDF file, 0.1 MB. Copyright © 2018 Yoshida et al. 2018 Yoshida et al. This content is distributed under the terms of the Creative Commons Attribution 4.0 International license.

10.1128/mSystems.00057-18.1 FIG S1 Sequences of the probes used for PS-TF screening. Download FIG S1, PDF file, 0.02 MB. Copyright © 2018 Yoshida et al. 2018 Yoshida et al. This content is distributed under the terms of the Creative Commons Attribution 4.0 International license.

Each of three DNA probes formed a single band at the position of the estimated size (see Fig. S1 for the probe sequences). TF-probe complexes exhibited different migration patterns, depending on the TF species: some formed TF-probe DNA complex bands, but the majority of TF-probe complexes formed a smear on PAGE or remained at the top of the gel (Fig. 2). The binding of test TFs to the promoter probes was then judged, relying on the disappearance of free unbound probes. In this first round of PS-TF screening, a total of 74 TF species (55 group A TFs and 19 group B TFs) were found to bind to both the *rsd* and *rmf* probes, although the binding affinities appeared different between these TFs (Fig. 2). In the mixed-PAGE system for PS-TF screening, however, some TFs exhibited significant level of binding to the unrelated probe added as an internal reference (Fig. 2). The considerable level of TF binding to this unrelated probe could be attributable to several reasons as summarized below (see Discussion).

**FIG 3:**
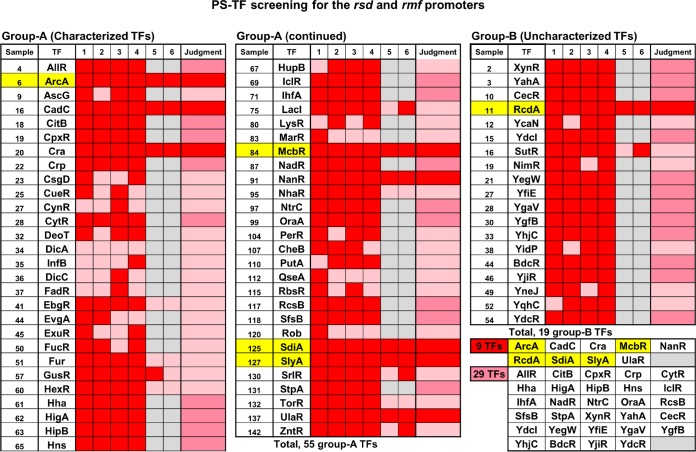
Summary of PS-TF screening for TFs with binding activity to both the *rsd* and *rmf* promoters. A total of 194 TFs were subjected to PS-TF screening. (The pattern of the first-cycle PAGE is shown in Fig. 2.) After four cycles of the screening, a total of 74 TFs (55 group A TFs and 19 group B TFs) were found to bind to both the *rsd* and *rmf* probes. These TFs were classified into two groups: TFs shown in red exhibited strong binding to both *rsd* and *rmf* probes, while TFs shown in pink exhibited weak or faint binding to the *rsd* and/or *rmf* promoter. A selected group of 19 stress response TFs (17 group A TFs and 2 group B TFs) with strong binding activity to both probes were further subjected to the fifth and sixth cycles of PS-TF screening, while 29 TFs that showed strong binding up to the fourth cycle were not subjected to the fourth and fifth cycles (shown in the box “29 TFs” in the bottom right corner). Taking all these results together, we identified the candidate TFs with strong binding to both the *rsd* and *rmf* probes as shown in the “Judgment” column. A set of 9 TFs showed strong binding to both probes for all six cycles of PS-TF screening (shown in the box “9 TF” included in the bottom right corner). TFs highlighted in yellow represent those analyzed in detail in this article.

Due to unavoidable fluctuation in gel patterns, we repeated the PS-TF screening for four cycles. As a result, the binding to both the *rsd* and *rmf* probes was weak for 26 TFs in at least one PAGE analysis, but a total of 48 TFs reproducibly showed binding activity to both the *rsd* and *rmf* probes (Fig. 3). To further focus the TFs to be employed to detailed analysis, we performed two more PS-TF cycles for a total of 19 TFs (17 group A TFs with known regulatory functions and 2 group B TFs, RcdA and SutR, of which the functions have been identified recently), mainly focusing on stress response TFs. As a result, 9 TFs (ArcA, CadC, Cra, McbR, NanR, RcdA, SdiA, SlyA, and UlaR) were identified to be positive for all six cycles of PS-TF screening (Fig. 3). The rest of the 27 TFs (20 known TFs and 7 unknown TFs) that exhibited strong binding to both probes up to the first 4 cycles of PS-TF screening were not subjected to the fifth and sixth screenings (Fig. 3).

In the course of gSELEX screening of regulatory targets of more than 200 TFs from E. coli, we already knew that a number of stress response promoters are generally under the control of a number of TFs, thus referred to as “multifactor promoters” (32), each sensing a specific environmental signal or condition (32). For instance, the promoter of the *csgD* gene encoding the master regulator of biofilm formation is under the control of more than 10 TFs (32, 40–42). The promoter of the *sdiA* gene for cell division control is also under the control of about 15 TFs (38). Likewise, the *rsd* and *rmf* promoters could also be multifactor promoters.

Besides this large set of TFs with binding activity to both *rsd* and *rmf* probes, a small number of TFs bound only to either the *rsd* or *rmf* probe. A total of 11 TFs (CueR, DmlR, GlpR, QseF, OmpR, UxuR, BtsR, YfeR, YhaJ, YiuA, and YijO) were estimated to bind only to the *rmf* promoter, while 2 TFs (DsdC and PaaX) were bound only to the *rsd* promoter (Fig. 2).

### Selection of a set of representative TFs recognizing both *rsd* and *rmf* promoters.

Since the anti-RpoD sigma Rsd and the ribosome dimerization factor RMF are both formed during the growth transition from exponential growth to the stationary phase, it is reasonable to assume that a common and the same regulation system operates between the two genes by employing the same set of TFs, which altogether sense a variety of unfavorable environmental conditions or factors. After six cycles of PS-TF screening (see Fig. 2 and 3), we selected five stress response TFs (ArcA, McbR, RcdA, SdiA, and SlyA) with binding activity to both the *rsd* and *rmf* promoter regions and subjected them to detailed analysis *in vitro* and *in vivo* of their effects on these two genes for transition from the growth phase into the stationary phase.

ArcA is a representative regulator for the switch of energy metabolism during the transition into the stationary phase, playing a key role in anoxic redox control through repression of a set of the operons involved in respiratory metabolism (43, 44) and activating the genes for fermentative metabolism (45). In the growth transition into the stationary phase, E. coli cells communicate with each other for coordinated transition for survival. Two quorum sensing (QS) signals for cell-cell communication are recognized by SdiA and McbR. SdiA senses a set of homoserine lactone (HSL) AI-1 (autoinducer-1) signals (35) and regulates transcription of the genes involved in cell division, motility, chemotaxis, and biofilm formation (46, 47), while McbR senses the cell-cell communication signal AI-2 (48) and regulates the formation of colonic acid and biofilm (49). In addition, two representative TFs, RcdA and SlyA, both participating in the control of biofilm formation were also selected for detailed analysis: RcdA is one of TFs involved in regulation of the *csgD* gene encoding the master regulator of biofilm formation (50), and SlyA is involved in control of biofilm formation through modulation of membrane lipopolysaccharides (51, 52).

### Binding sites of the five stress response TFs on the *rsd* and *rmf* promoters.

The five stress response and biofilm-inducing TFs all bound to both the 300-bp-long *rsd* promoter probe and 256-bp-long *rmf* promoter probe (see Fig. 2A, TF lanes 006 for ArcA, 084 for McbR, 125 for SdiA, and 127 for SlyA, and Fig. 2B, TF lane 011 for RcdA). In order to confirm the binding of these five stress response TFs to the *rsd* and *rmf* promoters and to map their binding regions on these two promoters, we constructed a set of divided segments starting from the respective full-size probes and examined the binding of five TFs onto each of the segment probes. Starting from the original 300-bp-long full-size *rsd* probes, a set of 10 smaller probes was prepared (Fig. 1A, panel A1), while starting from the 256-bp-long *rmf* probe, a set of 8 smaller probes were prepared (Fig. 1B, panel B1). All of these smaller promoter fragments formed a separate band on PAGE (Fig. 1A, panel A2, and Fig. 1B, panel B2, None lane). We then incubated these probe mixtures with each of the five test TFs. The DNA fragments containing the binding site for each TF disappeared from the original positions and migrated to probe-TF complex bands (Fig. 1A, panel A2, and Fig. 1B, panel B2). The binding regions of each TF were estimated based on the migration patterns (Fig. 1A, panel A3, and Fig. 1B, panel B3).

In the case of the *rsd* promoter, ArcA bound to fragments L5, R1, R2, and R3, and thus its binding site was predicted to be within a narrow sequence near 100 bp upstream from the Rsd-coding sequence. The known consensus sequence recognized by ArcA, 5′-(AT)GTTAATTA(AT)-3′ (45), exists within this region (Fig. 1A, panel 3, ArcA slot). SlyA also bound to a narrow region included in the promoter segments L5, L4, R1, R2, and R3 (Fig. 1A, panel A3, SlyA slot), suggesting a certain level of overlap of the SlyA-binding site with that of ArcA binding. On the other hand, most of the shorter fragments of the *rsd* probe disappeared for the other three TFs (McbR, RcdA, and SdiA) (Fig. 1A, panel A3), implying their binding to wide sequences approximately between −100 and −200 for McbR, between −120 and –220 for RcdA, and between −50 and −280 for SdiA. The wide-range binding of these TFs might indicate the binding of more than one TF molecule on the *rsd* promoter probe. Alternatively, this may suggest protein-protein cooperative binding, which was already observed for the binding of multiple RcdA molecules near its initial binding site (53).

We also examined the binding regions along the *rmf* promoter for these five TFs, using a total of 8 smaller segments of the *rmf* promoter (Fig. 1B, panel B2). The binding regions for the five TFs were then predicted on the basis of the gel pattern of TF-probe complexes (Fig. 1B, panel B3). The order of the size increase of TF-binding sequences was as follows: ArcA < SlyA < McbR < RcdA < SdiA. The sizes of binding sequences of these TFs were essentially the same as those observed with the *rsd* promoter. TFs with wide-range binding activity might be due to either the presence of multiple TF-binding sites on these probes or the cooperative protein-protein interaction along probe DNA as noted above (53).

### Influence of specific effectors on the activity of five stress response TFs.

Each of the five TFs herein examined is known to sense an external signal, as listed in Table 1, thereby controlling its activity and specificity ultimately leading to switch the growth-phase-dependent pattern of the genome expression. Under the anaerobic conditions, ArcA, the response regulator of the ArcAB two-component system (TCS), is phosphorylated by the ArcB sensor kinase (54), but can also be phosphorylated by high concentrations of acetyl phosphate (AcP) (55). Two known QS signals (AI-1 and AI-2) for cell-cell communications are recognized by SdiA and McbR, respectively, but the involvement of other TFs in QS signal recognition is not yet excluded. Previously we examined the recognition specificity of SdiA using a collection of AI-1 analogues and identified three species of AI-1 analogue that influenced the regulatory properties of SdiA (35). AI-2 stimulated the biofilm formation by E. coli through the motility regulator MqsR, which induces the expression of McbR (49), although the direct interaction of AI-2 with McbR has not yet been determined. RcdA was identified as one of the multiple regulators involved in regulation of the *csgD* gene encoding the master regulator of biofilm formation (40, 50). Previously we published the crystal structure of RcdA and its unique mode of DNA binding (53).

**TABLE 1:** Characteristics of five stress response TFs

TF	*M*_r_ (Da)	Family	Regulatory function(s)	Effector^a^	No. of targets^b^	Target TFs (total)
ArcA	27,292	OmpR	Anoxic redox control	AcP	89∼102	AbgT, ArgR, BetI, FeaR, GadE, GadX, GlcC, HcaR, LrhR, PdhR, RutR, UxpC, YdcI (13)
McbR	25,151	GntR	Biofilm formation (colonic acid production)	None	27∼36	CaiF, GadX, YbdO, YbeF (4)
				AI-2	26∼37	
RcdA	20,307	TetR	Biofilm formation (*csgD* regulation)	Acetate (pH 6)	31∼44	AdiA, AppY, CsgD, RcdA, StpA, Sxy, YgE (7)
SdiA	28,117	LuxR	Cell division and biofilm formation	None	48∼70	AbgR, DpiB, DsdC, LeuO, PdhR, SlyA, YdeF (7)
				HSL-A	77∼111	
				HSL-F	67∼97	
				HSL-K	57∼78	
SlyA	16,353	MarR	Biofilm formation (hemolysin synthesis)	ppGpp	10∼12	FlhD, GadX, IclR, LeuO, MurR, SdiA, SlyY, TreR, YlfB, YiiE (10)

a AI-1 (autoinducer 1) is represented by homoserine lactone (HSL) and HSL homologs (13). AI-2 (autoinducer 2) represents furanosyl borate diester. ppGpp (stringent response alarmone) represents guanosine tetraphosphate.

b The numbers of regulatory targets were determined by genomic SELEX screening (for details, see Fig. 8 and Table S3).

**FIG 4:**
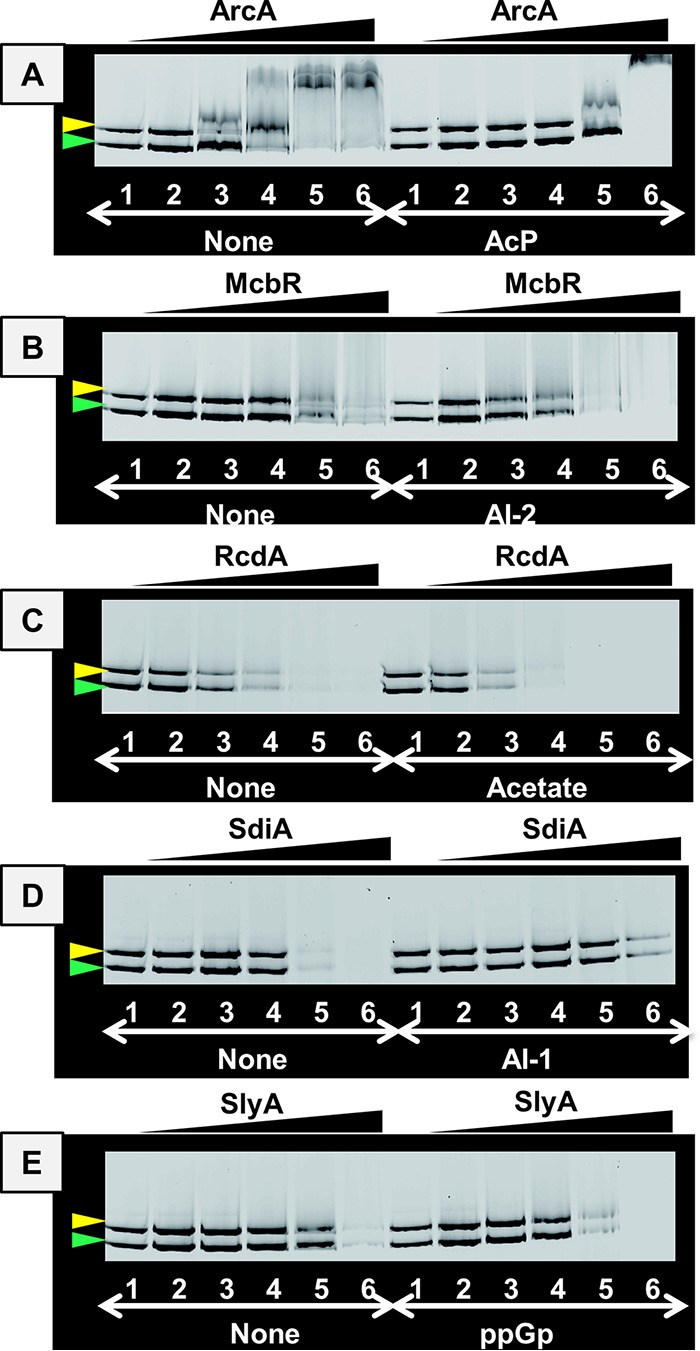
Influence of effectors on the DNA-binding activity of five TFs. A mixture of 0.5 pmol each of FITC-labeled *rsd* and *rmf* probes was incubated with increasing concentrations of TFs (lanes 1 to 6: 0, 0.5, 1, 2, 4, and 8 pmol, respectively) in the presence of 10 mM each of the following effectors: (A) AcP for ArcA, (B) AI-2 for McbR, (C) acetate for RcdA, (D) A-1 (normal HSL) for SdiA, and (E) ppGpp for SlyA.

Previously we determined the intracellular concentrations of TFs in E. coli K-12 W3110 (56). The level of ArcA showed a marked increase from about 100 molecules per genome in the exponential phase to about 240 in the stationary phase. The levels of the other four TFs, however, remained rather constant (see Table S3 in the supplemental material), indicating that the activity of these TFs could be controlled through interaction with effector ligands. We then tested the influence of the proposed signal molecule on each TF by analyzing the binding activity to the *rsd* and *rmf* promoters in the presence and absence of effectors. In the presence of increasing concentrations of test TFs, the level of TF binding to both the *rsd* and *rmf* promoter probes increased concomitantly with the increase of TF addition. The amounts of TF needed to bind to all the probes were different in the presence and absence of effectors as judged from the disappearance of free probe (Fig. 4). This finding indicates the DNA-binding activity of each TF changes when it bound to the signal molecule.

The binding to both the *rsd* and *rmf* probes decreased in the presence of effectors such as AcP for ArcA (Fig. 4A) and AI-1 for SdiA (Fig. 4D). In contrast, the binding to both probes increased in the presence of effectors such as AI-2 for McbR (Fig. 4B), acetate for RcdA (Fig. 4C), and ppGpp for SlyA (Fig. 4E). These findings support the prediction that the activity of all five TFs is controlled by specific effector ligands. To test possible influence of the observed effector responsibility of TFs on their regulatory functions, we next examined the roles of these TFs on expression *in vivo* of the *rsd* and *rmf* genes.

### Regulatory roles of five TFs on *rsd* and *rmf* gene expression: expression of *rsd* and *rmf* in the absence of TFs.

**FIG 5:**
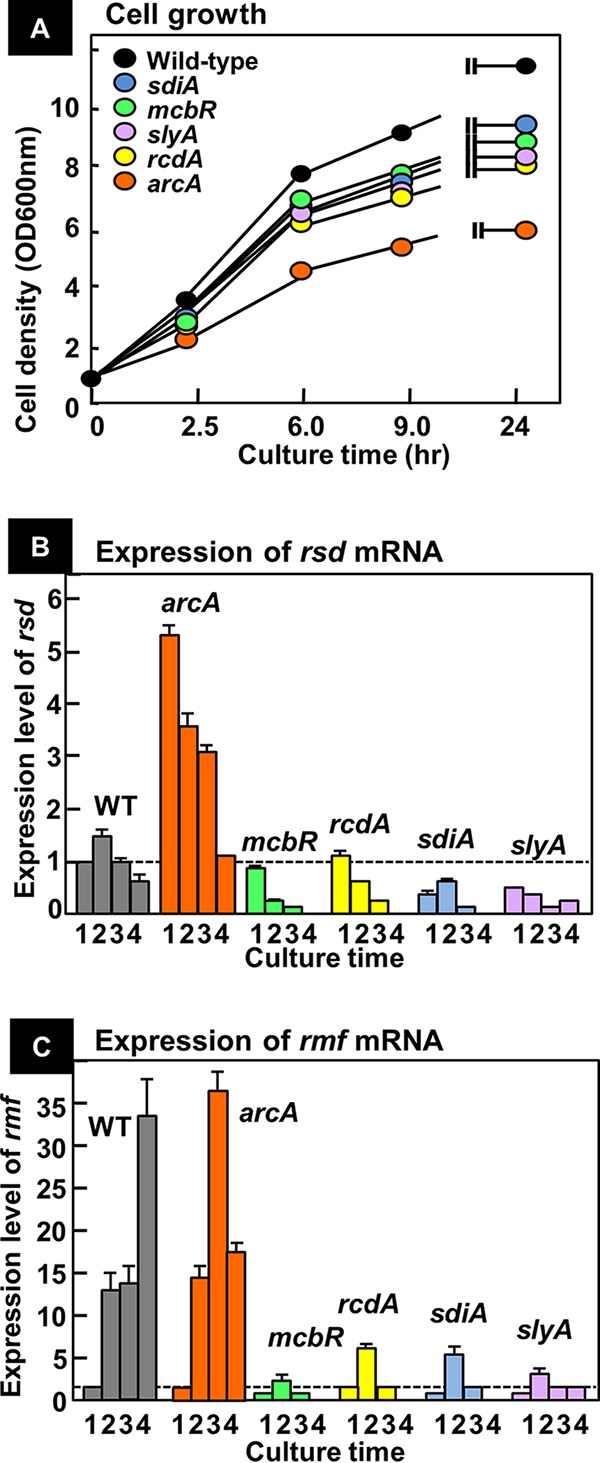
Influence of the lack of five TFs on the expression of *rsd* and *rmf* genes. (A) Wild-type and mutant strains, each defective in one of the five TF genes, were grown in medium E containing 2% polypeptone and 0.5% glucose, and growth was monitored by measuring cell density. (B) The level of *rsd* mRNA was measured by qRT-PCR at 2.5 (lane 1), 6.0 (lane 2), 9.0 (lane 3), and 24 (lane 4) h after inoculation of each strain. (C) The level of *rmf* mRNA was measured as in panel B. For both panels B and C, the level of mRNA is shown as the relative value to that at the 2.5-h culture of wild-type cells. The measurements were repeated three times, and each *P* value was calculated by using *C_T_* values of <0.05 for the wild-type and mutant strains.

In order to examine possible roles of five selected TFs on the expression of *rsd* and *rmf* genes, we first examined the influence of deletion of each of the genes encoding ArcA, McbR, RcdA, SdiA, and SlyA. When these five mutants are grown in medium E containing 2% polypeptone and 0.5% glucose, the growth of all of these mutants was retarded (Fig. 5A). The growth rates of *rcdA*, *sdiA*, *mcbB*, and *slyA* mutants in the exponential phase were approximately half of that of the wild type (WT), but the growth rate of the *arcA* mutant was less than 30% of that of the wild type, suggesting the requirement of ArcA for normal growth. After a 24-h culture, the level of cell growth was less than 75% for all these mutants, and the growth retardation was maximum (less than 50% of the wild-type level) for the mutant lacking *arcA.* These findings indicate the involvement of all five TFs for normal growth of E. coli K-12 W3110. In particular, ArcA was needed for growth even during exponential growth.

Under the same culture conditions and at various times, we then measured the levels of mRNA for the *rsd* and *rmf* genes by using quantitative real-time PCR (qRT-PCR). In wild-type E. coli, the level of *rsd* mRNA increased transiently during the growth transition from exponential to stationary phase (Fig. 5B). This finding agrees well with the pattern of the Rsd protein level upon entry to the stationary phase of E. coli K-12 W3110 (57). Next we measured the level of *rsd* mRNA for all five TF mutants. In the *arcA*-defective mutant, the level was severalfold higher than that of the wild type (Fig. 5B), suggesting the repression of Rsd expression by ArcA in the exponentially growing cells. Under hypoxia conditions upon entry into the stationary phase, however, this repression apparently disappeared—supposedly because ArcA repressor might be inactivated through phosphorylation by sensor kinase ArcB under a defective supply of oxygen. On the other hand, the level of *rsd* mRNA in the other four TF mutants (*mcbR*, *rcdA*, *sdiA*, and *slyA*) was significantly lower than that of the wild type (Fig. 5B), implying that these four stress response TFs are involved in transcription activation of the *rsd* gene.

Using the same cultures, we also measured the level of *rmf* mRNA. In wild-type E. coli K-12, the level of *rmf* mRNA markedly increased concomitant with the growth transition from exponential phase to stationary phase, ultimately reaching a level more than 30-fold higher than that of the wild type (Fig. 5C). This finding agrees well the growth-phase-dependent synthesis of RMF protein in E. coli K-12 W3110 (16, 21). The pattern of growth-dependent variation of *rmf* mRNA in the mutant lacking the *arcA* gene was essentially the same as that of the wild type (Fig. 5C). In contrast, the levels of *rmf* mRNA were markedly decreased for the other four mutants lacking McbR, RcdA, SdiA, or SlyA (Fig. 5C). The levels of influence of gene knockout of the five TFs tested in this study were essentially the same between the *rsd* and *rmf* genes, indicating that these four TFs are involved in transcription activation of the two genes *rsd* and *rmf*, together playing key roles in growth-phase-coupled switching in genome expression from exponential growth to stationary phase.

### Regulatory roles of five TFs on the *rsd* and *rmf* gene expression: expression of *rsd* and *rmf* after overexpression of TFs.

**FIG 6:**
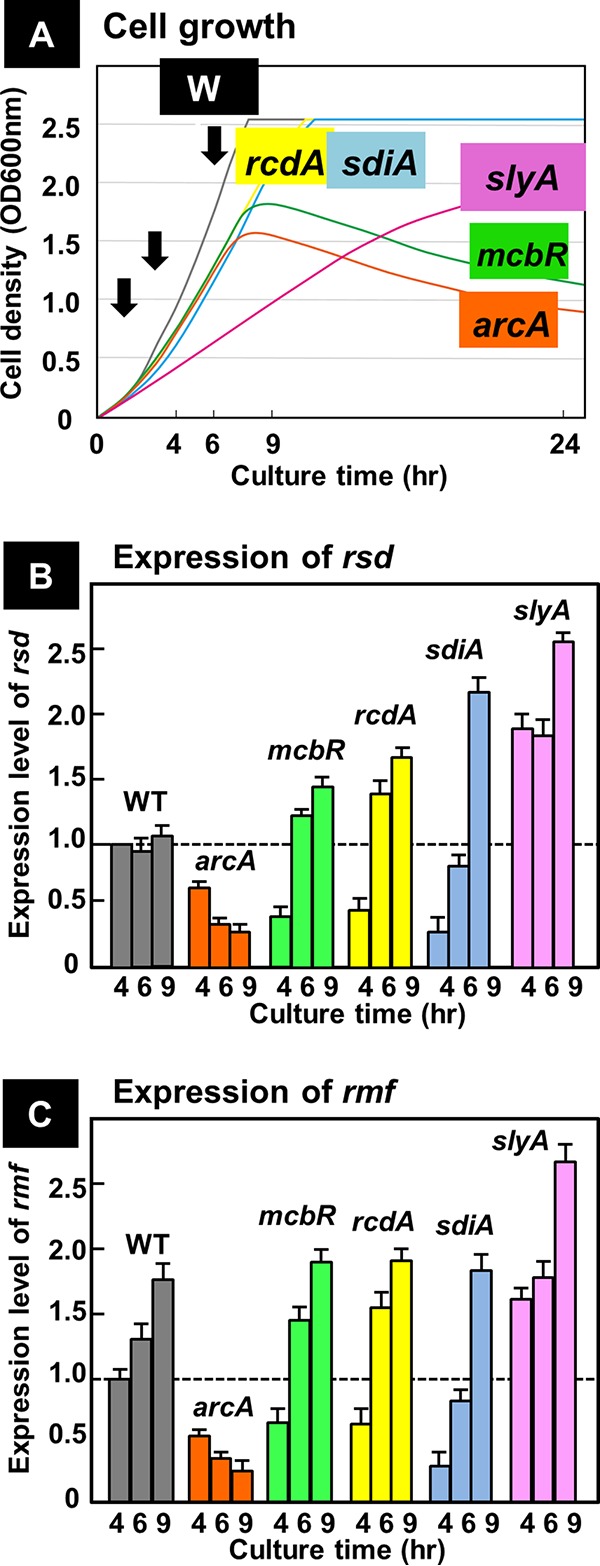
Influence of the overexpression of five TFs on the expression of *rsd* and *rmf* genes. (A) The wild type was transformed with each of five TF expression plasmids. The wild-type and transformed cells were grown in LB medium. Cell growth was monitored by measuring the cell density. At 3 h after inoculation of overnight culture into fresh medium, 50 µM IPTG was added for induction of TF expression. RNA samples were taken at 1, 3, and 6 h after induction for measurement of mRNA for each TF. (B) The level of *rsd* mRNA was measured by using qRT-PCR at the three growth phases indicated by arrows in panel A. (C) The level of *rmf* mRNA was measured as in panel B. For both panels B and C, the level of mRNA is shown as the relative value to that at the 4-h culture of wild-type cells. The measurements were repeated three times, and each *P* value was calculated by using *C_T_* values of <0.05 for the wild-type and mutant strains.

To confirm the involvement of the five stress response TFs in transcription regulation of the *rsd* and *rmf* genes, we next tested the possible influence of overexpression of these TFs. First, we examined the influence of TF overexpression on cell growth. As in the case of deletion of TF genes, the cell growth was more or less retarded after overexpression of each of these five TFs, but in different manners (Fig. 6A). One unexpected influence was the marked decrease of cell growth rate for the *slyA* mutant, exhibiting more than a 2-fold decrease in growth rate compared with the wild-type parent (Fig. 6A; *slyA*), implying that overexpression of SlyA interferes with normal growth of E. coli K-12. In contrast, the growth rates of TF-expressing cells were essentially the same between the other four TFs (RcdA, SdiA, McbR, and ArcA) at about half the level of the wild type, but after the late log phase, the growth of cells overexpressing *mcbR* and *arcA* suddenly stopped, and afterward the cells showed a gradual decrease in cell density (Fig. 6A; *mcbR* and *arcA*), indicating the requirement of McbR and ArcA for survival upon entry into the stationary phase.

Under these TF-expressing conditions, we measured the level of *rsd* and *rmf* mRNA by qRT-PCR. In the wild-type E. coli, the level of *rsd* mRNA stays rather constant throughout growth transition (Fig. 6B; WT). The *rsd* mRNA markedly decreased in cells overexpressing ArcA (Fig. 6B; ArcA). In contrast, *rsd* mRNA increased significantly at the late phase of cell growth in cells overexpressing McbR, RcdA, SdiA, and SlyA (Fig. 6B). The variation pattern of *rsd* mRNA was completely opposite to that observed in the absence of these five TFs (Fig. 5B). Taken together, we concluded that ArcA represses transcription of the *rsd* gene, while McbR, RcdA, SdiA, and SlyA activate its transcription. The level of *rsd* mRNA in cells overexpressing SlyA increased even in the exponentially growing cells (Fig. 6B; SlyA), in agreement with the growth retardation of SlyA-expressing cells even in the log phase (Fig. 6A; SlyA).

Next we examined the level of *rmf* mRNA in TF-overexpressing cells. The level of *rmf* mRNA decreased in cells overexpressing ArcA, but increased when other four TFs (McbR, RcdA, SdiA, and SlyA) were overexpressed (Fig. 6C). The levels of influence of TF overexpression were essentially the same between the *rsd* and *rmf* gene. The high-level induction of *rmf* mRNA throughout the growth phase in the cells overexpressing SlyA was essentially the same with the pattern of *rsd* mRNA. Taken altogether, we conclude that the *rsd* and *rmf* genes are both under the same regulation network involving the five TFs tested in this study.

### Level of functional Rsd in the absence of five stress response TFs.

In the absence of repressor ArcA, the level of mRNA for anti-sigma Rsd increased, while it decreased in the absence of activators, McbR, RcdA, SdiA and SlyA (Fig. 5B). We then tried to measure the level of Rsd protein in the absence of these TFs. For this purpose, His-tagged RpoD sigma factor was highly expressed, which should associate free unused anti-sigma Rsd for trapping (14, 57). His-tagged RpoD was affinity isolated, and Rsd protein recovered in this complex was quantitated by immunoblotting.

**FIG 7:**
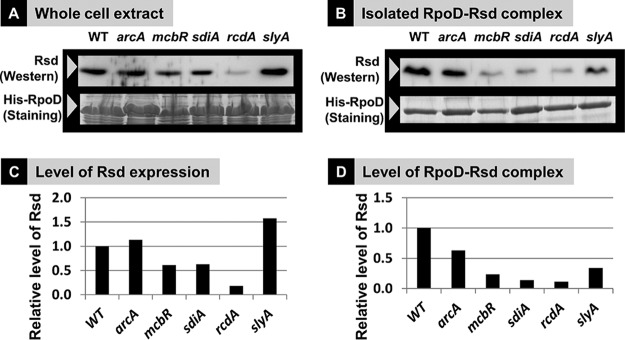
Influence of the lack of five TFs on the expression of Rsd protein. (A) The wild type and five mutants, each lacking the indicated gene on top, were grown in medium E containing 2% polypeptone and 0.5% glucose. Cells were harvested in the early stationary phase. The total amount of Rsd in whole-cell extract was measured by Western blotting, while the amount of affinity-purified His-tagged RpoD was measured by protein staining with Coomassie brilliant blue (CBB). The level of Rsd protein, shown in panel C, was determined by densitometry of the gel pattern and is shown as the relative value to that in wild-type cells. (B) His-tagged RpoD-Rsd complexes were affinity purified. The amount of Rsd bound on this complex was measured by immunoblotting. The total amount of His-tagged RpoD was observed by protein staining with CBB. The accuracy of immunoblot measurement by the method herein employed is more than 90% (56).

The level of Rsd protein in whole-cell extracts significantly decreased for the cells lacking the activator TFs (McbR, SdiA, and RcdA) (Fig. 7A and C) but increased in the absence of activator SlyA (Fig. 7A and C, *slyA* lane). The level of Rsd protein stayed unaltered in the presence and absence of repressor ArcA (Fig. 7A and C, *arcA* lane). Comparing the levels of mRNA (Fig. 5) and protein (Fig. 7), a considerable difference exists for the mutants lacking ArcA and SlyA.

This difference might be due to difference in translation efficiency of *rsd* mRNA in the *arcA* and *slyA* mutants. The level of RpoD-associated Rsd was then measured after affinity purification of His-tagged RpoD-Rsd complexes. The amount of RpoD-bound Rsd was decreased for cells lacking the activators McbR, SdiA, RcdA, and SlyA (Fig. 7B and D), even though a certain level of difference was observed between these mutants with respect to the amount of RpoD-bound Rsd relative to the total Rsd protein. This finding indicates the involvement of as yet unidentified factors in the formation of RpoD-Rsd complex.

### Level of functional RMF in the absence of five stress response TFs: estimation of the 100S ribosome level.

**FIG 8:**
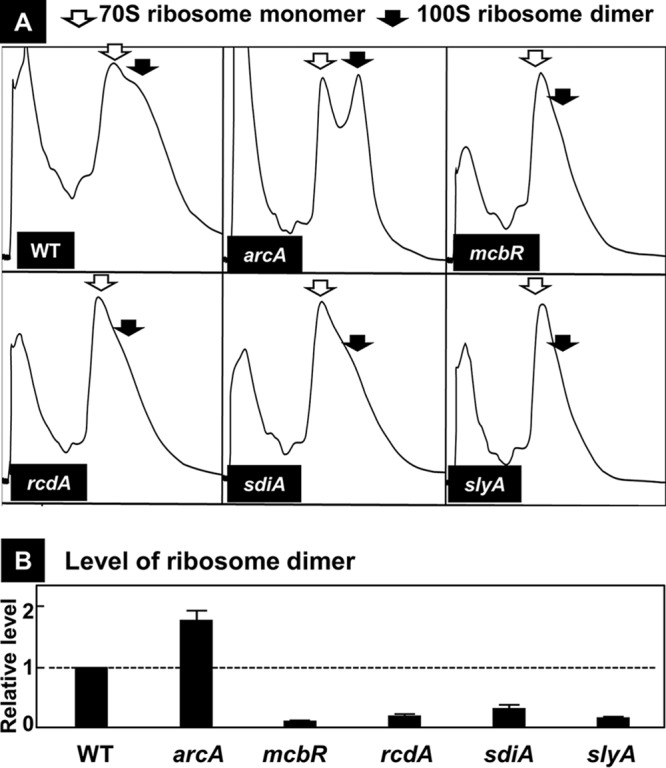
Influence of the lack of five TFs on the formation of 100S ribosome dimers. (A) The wild-type and mutants, each lacking one of the five TF genes, were grown in medium E containing 2% polypeptone and 0.5% glucose, and cells were harvested in the early stationary phase. Cell lysates were subjected to sucrose gradient centrifugation for monitoring of the ribosome patterns. Open arrows indicate 70S ribosomes, while filled arrows indicate 100S dimers. The experiments were repeated 3 times. (B) The level of 100S ribosomes was estimated by using Systat software for peak separation analysis (Systat Software, Inc., Japan). The measurements were repeated three times, and each *P* value was calculated by using the quantitative ratios of 70S and 100S ribosomes of <0.05 for the wild-type and mutant strains.

RMF binds to 70S ribosomes, thereby converting ribosomes into inactive 100S dimers for translational repression (16, 21). Measurement of *rmf* mRNA by qRT-PCR indicated that the level of RMF increases in the *arcA* mutant, but decreases in the *mcbR*, *rcdA*, *sdiA*, and *slyA* mutants (Fig. 5C). We then examined whether the observed alteration in RMF level influences the formation of 100S ribosomes. Cell extracts from the wild type and five TF mutants were analyzed by sucrose density gradient centrifugation. The level of 100S ribosome dimers increased in the middle of the transition phase from exponential phase to stationary phase (Fig. 8A). The level of 100S dimers was significantly higher for the mutant lacking the *arcA* gene (Fig. 8A), in agreement with the increase in RMF in this particular mutant (Fig. 5C). In contrast, the amount of 100S ribosomes was significantly lower for other mutants lacking McbR, RcdA, SdiA, and SlyA (Fig. 8A).

The level of 100S ribosome dimers as measured by sucrose gradient centrifugation also supported the observed alteration of the 100S dimer level (Fig. 8B). Taken together, we concluded that the pattern of 100S ribosome dimers agreed well with the intracellular level of RMF protein.

### Whole sets of the regulatory targets of five stress response TFs.

**FIG 9:**
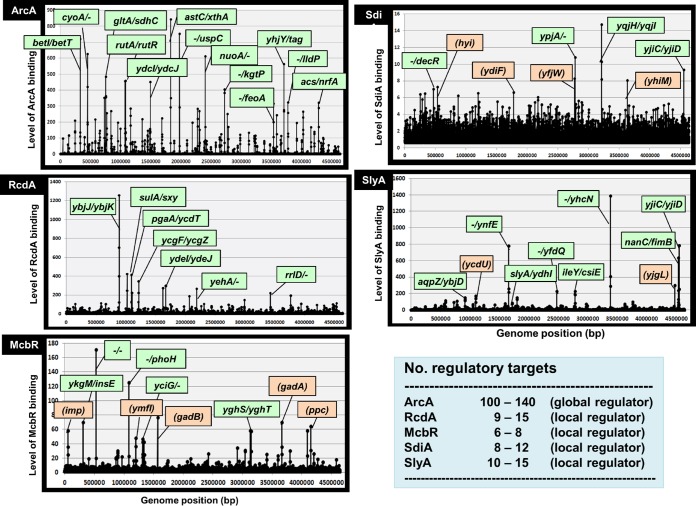
Genomic SELEX screening of regulatory targets of five TFs. gSELEX screening of regulatory targets was performed using each of the five purified TFs. The gSELEX pattern was analyzed by using a tiling array as described in Materials and Methods. The total number of regulatory targets was estimated by setting similar cutoff levels for all patterns. The list of possible targets for each TF is shown in Table S4.

The five stress response TFs examined herein were found together to regulate the key players Rsd and RMF involved in the level of control of the transcriptional apparatus and translational machinery during the growth transition into the stationary phase. This finding raises the possibility that these regulators could be involved in regulation of genes other than the *rsd* and *rmf* genes. We then tried to identify the whole set of regulatory targets of these five TFs by using gSELEX screening (58, 59). After six cycles of gSELEX screening, TF-bound DNA segments were analyzed with use of a tiling array system (58, 60). All five TFs gave clean gSELEX patterns (Fig. 9), from which the regulatory targets were estimated. Since we repeated in this study the gSELEX screening for six cycles, TFs with high affinity to each probe could be detected, but low-affinity probes might be lost concomitant with the repetition of the SELEX cycle. As to these five TFs, we detected only low-level peaks at the *rsd* and *rmf* promoter regions after six cycles of gSELEX, even though they bound to the two promoter regions at a single round of PAGE assay, as noted above (Fig. 4).

ArcA was suggested to repress the *rsd* and *rmf* promoters during the growth phase under sufficient supply of oxygen, thereby preventing the synthesis of anti-sigma Rsd and ribosome dimerization factor RMF. Upon activation of ArcA by phosphorylation, this repression should be released, but instead the activated ArcA gave a total of about 70 new binding peaks (Fig. 9, ArcA panel). Based on the position of ArcA binding, we estimated about 100 regulatory targets (Table 1; for details, see Table S4A in the supplemental material), indicating that ArcA could be classified as one of the global regulators (for the TF classification, see reference 31). The majority of regulatory targets of ArcA are the genes involved in energy metabolism, in particular, under anaerobic conditions (for details see Discussion and Table S4A). In addition, a set of stress response genes for the modification of cell surface components were detected, such as transporters of some carbon sources (CaiT, BetT, DcuC, KdpE, Fiu, UgpB, and FeoA), uptake of external iron (*feoA* for ferrous iron transporter and *fiu* for siderophore transporter), and cell adhesion (Elf, YehD, YcbQ, and YcgR). Since as many as 13 species of TFs are under the direct control of ArcA, a large group of stress response genes should be indirectly regulated by ArcA (for details, see Table S4A).

Under unfavorable growth conditions, bacteria communicate with each other for collaboration for adaption and survival. The involvement of two kinds of quorum sensing (QS) signal, AI-1 (*N*-acyl-homoserine lactone-type QS) and AI-2 (dihydroxypentane-2,3-dione [DPD]-type QS), has been established for cell-cell communication for E. coli (61, 62). SdiA plays a role in sensing AI-1 (63), while McbR participates in recognition of AI-2 (48). Besides the normal AI-1, three kinds of HSL analog were found to interact with SdiA (35). Here we identified the involvement of both SdiA and McbR in regulation of both the *rsd* and *rmf* genes. After gSELEX screening, the whole set of regulatory targets were estimated for both SdiA and MabR (Fig. 9, McbR and SdiA panels). A total of 70 to 110 targets were identified for SdiA in the presence of normal HSL and each of three kinds of HSL analogs (Fig. 9, SdiA panel, and Table 1; for details, see Table S4D). Depending on the effector species, the target selection patterns differed to various extents between 4 effectors, with overlap of 20 to 30%. Thus, SdiA is a good example of the control of target selectivity by effector ligands. The regulatory targets of SdiA include varieties of transporters such as C-dicarboxylate (DcuC), formate (FocA), aromatic amino acids (AroP), shikimate (ShiA), iron (Fiu), and Ca^2+^/Na^+^:proton antiporter (ChaA). Seven TFs are also under the control of SdiA (Table 1), indicating indirect regulation of a large set of targets. In the case of AI-2 sensing McbR, the recognition of 30 to 40 targets markedly differed in the presence and absence of AI-2 (Table 1; for details see Table S4B), including 4 transporters (CodB for cytosine, ProV for glycine betaine, FeoA for ferrous iron, and LivJ for leucine/isoleucine) and 4 TFs (CaiF, GadX, YbdO, and YbeF) (Fig. 9, McbR panel, and Table 1; for details see Table S4B).

Both RcdA and SlyA are known to influence biofilm formation. RcdA was identified as a regulator of the *csgD* gene encoding the master regulator of biofilm formation (50). A total of about 40 binding sites and about 30 regulatory targets were identified for RcdA (Fig. 9, RcdA panel; for details see Table S4C). The predicted targets are mostly related to the genes for stress response, including a set of membrane-associated proteins (CsgB, NanC, OmpA, PgaA, YbjJ, YehA, and YoeA) and cytoplasmic stress response proteins (Asr and YdeI). In addition, seven stress response TFs are also under the control of RcdA (Table 1), indicating the indirect regulation by RcdA of a number of genes under the direct control of these downstream TFs. RcdA exhibits strong cooperative DNA binding and produces aggregates of RcdA-DNA complexes (53).

SlyA was originally identified as a hemolytic protein in Salmonella (64), but little is known about the regulatory function of SlyA. Here we identified a total of more than 50 binding sites and about 80 regulatory targets on the E. coli K-12 genome by E. coli SlyA (Fig. 9, SlyA panel; for details see Table S4E). Here we found that SlyA plays an as yet unidentified important role in cell growth because when SlyA is overexpressed, the rate of cell growth decreases to less than half the level of the wild type (Fig. 6A). One unique feature is its binding to as many as 10 TF genes (Table 1), including the gene coding for LeuO, which is an essential global regulator of amino acid metabolism and a key antisensor against a number of metabolic genes that are repressed by the H-NS silencer (65). SlyA binding was also detected in the *fecI* gene encoding the minor sigma factor of RNAP.

**FIG 10:**
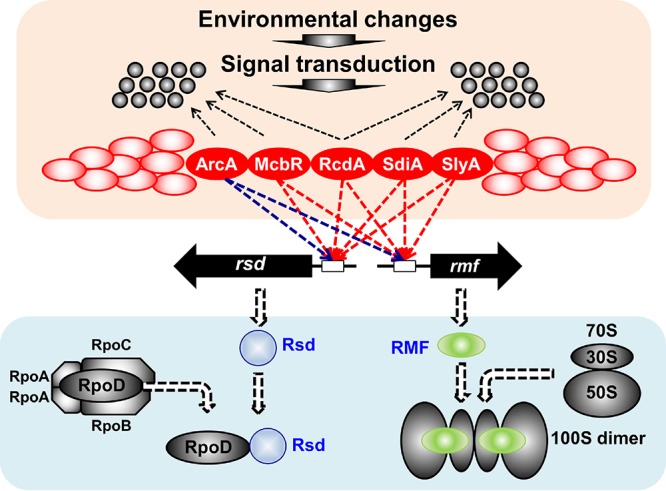
Regulatory roles of the five stress response TFs. After PS-TF screening, a number of TFs (shown in red circles) were suggested to regulate both the *rsd* and *rmf* genes. Detailed analyses of the regulatory roles *in vitro* and *in vivo* were performed for the five representative stress response TFs (ArcA, McbR, RcdA, SdiA, and SlyA). ArcA was indicated to repress transcription of both *rsd* and *rmf* genes (shown by blue dotted arrows), while other four TFs were suggested to activate both genes (shown by red dotted arrows). gSELEX indicated that all of these TFs regulate not only the *rsd* and *rmf* genes but also a number of genes (shown in small black circles) supposedly required for survival under stressful conditions.

The results of gSELEX screening indicate that the five TFs exhibit two modes of regulation: one for overall reduction of genome expression through functional switching of unused RNAP RpoD sigma factor and ribosomes into inactive forms for storage, leading to the coordinated hibernation of the transcriptional and translational apparatus, and another for control of the utilization pattern of the decreased amount of functional RNAP for induction of a specific set of genes for survival under stressful conditions, including biofilm formation (Fig. 10). It is noteworthy that each of these five TFs controls a set of TF genes from 4 up to 13 targets (Table 1), altogether forming large TF networks in which these five TFs are located upstream in the hierarchy.

## DISCUSSION

### PS-TF screening *in vitro* for the regulatory targets.

Wide varieties of modern biotechnology methods have been developed for global identification of the genome regulation. For instance, the identification *in vivo* of regulatory targets of TFs has been achieved by analysis of a whole set of transcripts by using microarrays and by analysis of the distribution of the transcriptional apparatus along the genome by using chromatin immunoprecipitation with microarray technology (ChIP-chip) or ChIP-DNA sequencing (ChIP-Seq) analyses (66–68). However, it is impossible to identify the whole set of regulatory targets *in vivo*, because (i) the regulatory proteins are not always expressed in E. coli cells (56), (ii) some regulatory proteins are not always functional, but their activities are controlled by protein modification, such as phosphorylation and acetylation or through interaction with effector ligands of small molecules (32), (iii) some regulatory proteins function in collaboration with other proteins, forming hetero-complexes (TEC database [www.shigen.nig.ac.jp/ecoli/tec/]), (iv) a set of regulatory proteins involved in regulation of a single and the same promoter compete with each other for binding to the overlapping DNA sites (41, 42), and (v) for modeling the genome-wide regulation with use of *in vivo* data, a single strain and the same E. coli strain must be used because a high-level variation exists in the gene composition and organization between E. coli strains, including the set of regulatory proteins for gene expression (69, 70). To overcome such problems associated with *in vivo* analysis of transcription, we have developed two *in vitro* systems: (i) gSELEX screening of regulatory target promoters, genes, and operons by a specific regulatory protein (32, 59) and (ii) PS-TF screening for search of TFs involved in regulation of a specific promoter (see reference 32 and this report). We have successfully employed gSELEX screening for more than 200 TFs from the same E. coli strain, K-12 W3110 (32). The gSELEX screening system is, in particular, useful as a shortcut approach for identification of the regulatory targets of hitherto uncharacterized TFs because in the case of E. coli, TFs generally bind near the promoters of their target genes, thereby allowing quick prediction of their regulatory targets. In fact, the regulatory functions have been identified for more than 10 uncharacterized TFs using the gSELEX system (32, 58).

In parallel with the gSELEX screening *in vitro* of regulatory target promoters, genes, and operons, we developed PS-TF screening *in vitro* of TFs involved in regulation of specific promoters. Even though the laborious work of TF purification is needed for application of this experimental system, the TF collection, once established, could be used for identification of the whole set of TFs for any promoter on the E. coli genome. Using the PS-TF screening system, we have already identified as many as 15 TFs for the promoter of the *sdiA* gene encoding the key regulator of cell division and cell-cell communication (38). Under a fixed standard reaction condition of the PS-TF screening herein employed, a considerable level of fluctuation was, however, observed because (i) the optimum conditions for TF binding to target DNA could be different between TFs, (ii) the optimum mixing ratios of TF and probe could be different between TFs, depending on the binding affinity to DNA probes, (iii) some TFs require effector ligands for expression of the activity, and (iv) a certain level of denaturation of purified TFs is unavoidable after prolonged storage. In order to prepare the regulatory proteins in functional forms at present, a systematic search for effectors affecting TF activities is in progress by our research team, including screening *in vitro* of natural and synthetic chemical compounds affecting the DNA-binding activity of TFs (35). The phenotype microarray (PM) system is one selection tool for screening *in vivo* of effectors affecting the functions of TFs (71, 72).

In this study, we performed a systematic application of the PS-TF screening, for the first time, for identification of TFs involved in regulation of the *rsd* and *rmf* promoters. Starting from a total of 74 candidate TFs obtained after the first screening, we selected 5 TFs (ArcA, McbR, RcdA, SdiA, and SlyA) after 6 cycles of screening for experimental confirmation of their participation in regulation *in vitro* and *in vivo* of the *rsd* and *rmf* promoters. Taken together with the gSELEX screening of the whole set of regulatory targets by these 5 TFs, we propose a model, shown in Fig. 10, that during the growth transition of E. coli K-12 from exponential growth to the stationary phase, a number of TFs, each sensing different environmental factors or conditions, participate in the coordinated and simultaneous regulation of the synthesis of Rsd and RMF, both of which are involved in the conversion of key components of the gene expression into inactive storage forms for hibernation.

In addition to a set of TFs for coordinated regulation of the *rsd* and *rmf* genes, we identified 11 TFs that bind only the *rmf* probe and at least 2 TFs that bind only to the *rsd* probe. The regulatory modes of these TFs specific for the synthesis of only Rsd or RMF await further studies.

### Regulatory roles of ArcA, McbR, RcdA, SdiA, and SlyA.

Upon entry into the stationary phase, the pattern of genome expression changes markedly through two modes of control of the gene expression apparatus: (i) the decreased level of both parts of the transcriptional and translational apparatus and (ii) the modulation of activity and specificity of the transcription and translation apparatus (for instance, see references 10 and 73). Based on all the results herein described, we propose that the five TFs (ArcA, McbR, RcdA, SdiA, and SlyA) examined in this study play dual roles in control of these two processes (Fig. 10).

Upon entry into the stationary phase, the synthesis of RNAP core enzyme and ribosomes is turned off, leading to the decrease in the overall capacity of gene expression. The major regulator for this switch is the stringent response alarmone (p)ppGpp, which is induced in the absence of nutrient supply. It binds to either the RpoZ omega subunit of RNAP (27) or RNAP-associated DksA (28), resulting in the decreased transcription of a set of genes encoding components of RNAP core enzyme, ribosomes, and tRNAs.

Besides this control of expression level, the preexisting RNAP and ribosomes are converted into inactive forms through induction of anti-sigma Rsd and ribosome dimerization factor RMF. Here we have identified, for the first time, the coordinated regulation of *rsd* and *rmf* genes by at least five representative TFs (ArcA, McbR, RcdA, SdiA, and SlyA). ArcA, the response regulator of the ArcBA TCS, is a well-characterized global regulator that regulates a large number of genes for metabolism in response to the lack of oxygen supply (74). The oxidized forms of quinone electron carriers in the membrane inhibit the autophosphorylation of ArcB sensor kinase, while under anaerobic conditions ArcB undergoes autophosphorylation and then phosphorylates ArcA (74). The activated ArcA represses a set of genes involved in respiratory metabolism, encoding enzymes for the tricarboxylic acid cycle, glyoxylate shunt, and fatty acid degradation (43, 44), while some genes encoding the enzymes for fermentative metabolism are activated (45, 75). The gSELEX screening herein described indicated more than 70% of its targets are indeed related to the metabolism of energy production under anaerobic conditions. In addition, we found the involvement of phosphorylated ArcA in regulation of a set of genes for survival response under stressful conditions as noted above.

Under unfavorable growth conditions, E. coli cells collaborate for adaption and survival through cell-cell communications via varieties of signal molecules. SdiA plays a role in sensing HSL (*N*-acyl-homoserine lactone)-type QS signal AI-1 (63). SdiA was originally identified as the “suppressor of the cell division inhibitor” that controls transcription of the *ftsQAZ* operon involved in cell division (47, 76, 77). By using a collection of 477 species of the chemically synthesized HSL analogues, we identified three “synthetic signal molecules” (SSMs) that bind to SdiA and modulate its recognition specificity of regulatory targets (38). In response to HSL, SdiA controls biofilm formation, motility control, antibiotic sensitivity, and virulence expression (46, 78, 79). Here we identified the involvement of SdiA in regulation of the *rsd* and *rmf* promoters. In addition, about 50 to 100 genes were found to be under the control of SdiA, including 7 stress response TF genes (Table 1; for details see Table S4D). On the other hand, the interspecies QS signal AI-2 is recognized by McbR, which also regulates biofilm formation and mucoidity through repressing the expression of periplasmic antitoxin McbA (48, 80, 81). MqsR links QS signal AI-2 to biofilm formation (82). MqsR is also highly expressed in persister cells, a subpopulation of genetically identical quiescent cells that exhibit multidrug tolerance (83). Here we identified the involvement of McbR in coordinated regulation of both the *rsd* and *rmf* genes.

Biofilm formation is also controlled by the newly identified proteins RcdA (renamed YbjK) and SlyA (E. coli homolog of Salmonella SlyA). RcdA was identified as a regulator of the *csgD* gene encoding the master regulator of biofilm formation in E. coli K-12 (50). The genomic SELEX screening indicated that RcdA is involved in regulation of 30 to 40 stress response genes (Table 1; Table S4C), including seven stress response TFs. This indicates that a large number of genes must be regulated indirectly via these TFs, which are under direct control by RcdA. The MarR family protein SlyA regulates the expression of cytolysin HlyE through antagonistic interplay with the general silencer of H-NS (84, 85). The well-characterized antisilencer LeuO (65) was also found to be under the control of SlyA (Table 1; Table S4E). SlyA also regulates the genes enhancing lipid A modification for biofilm formation (52). Here we identified the involvement of SlyA in regulation of the *rsd* and *rmf* genes for hibernation of the gene expression apparatus in the stationary phase. The regulatory role of SlyA further expands through control of as many as 10 TF genes, including the *sdiA* gene and the gene encoding the extracytoplasmic function (ECF)-type minor sigma factor FecI, which is required for transcription of the *fecABCDE* operon for ferric citrate transport (86). These results altogether indicate that SlyA is located upstream of the proposed TF network in E. coli K-12 (10, 32).

In conclusion, we propose dual regulatory roles for all 5 TFs during the growth transition from exponential growth to the stationary phase: one for controlling the level of the genome expression apparatus and another for controlling utilization of the transcriptional apparatus so as to express a set of genes for adaptation and survival under stressful conditions (Fig. 10).

## MATERIALS AND METHODS

### Bacterial strains and growth conditions.

The genome of E. coli K-12 W3110 type A (87) was used as the source of construction of TF expression plasmids, the DNA probes for PS-TF screening, and the DNA library for gSELEX screening of regulatory targets of TFs. E. coli DH5a was used for amplification of TF expression plasmids. E. coli BL21 was used for overproduction of all TFs used in this study. E. coli K-12 BW25113 [genotype F^−^ Δ(*araD-araB*)*567* Δ*lacZ4787*(::*rrnB-3*) λ^−^ *rph-1* Δ(*rhaD-rhaB*)*568 hsdR514*] and its mutant strains with *mcbR*, *rcdA*, *sdiA*, or *slyA* deleted were obtained from the Keio collection (88) of the E. coli Stock Center (National Bio-Resource Center, Mishima, Japan). The *arcA*-deleted mutant strain of BW25113 was constructed by replacing the gene with a kanamycin cassette as used for construction of the Keio collection. The deletion of the *arcA* gene was confirmed by PCR. For TF overexpression, E. coli K-12 strain AG1 [genotype *recA1 endA1 gyrA96 thi-1 hsdR17*(r_K_^−^m_K_^+^) *supE44 relA1*] was transformed with each of the TF expression plasmids from the ASKA clone library (89) that were obtained from the E. coli Stock Center (National Bio-Resource Center, Mishima, Japan).

For TF overexpression, E. coli cells were grown in Luria broth (LB) at 37°C with shaking. For analysis of ribosome profiling, cells were grown 37°C with shaking at 120 rpm in medium E containing 2% polypeptone and 0.5% glucose (90). Cell growth was monitored by measuring the optical density at 600 nm (OD_600_).

### Preparation of purified TF stocks.

Expression plasmids of all TFs were constructed according to the standard procedure (55). In brief, the TF coding sequences were PCR amplified using the E. coli K-12 W3110 type A genome DNA as a template and inserted into pET21α vector. Expression of a total of more than 200 His-tagged TFs was performed in E. coli BL21. His-tagged TFs were affinity purified according to the standard procedure (40, 55, 59). In brief, the transformants carrying each of TF expression plasmids were grown in LB medium up to an OD_600_ of 0.6 to ∼0.7, and then 1 mM IPTG (isopropyl-β-d-thiogalactopyranoside) was added for induction of TF expression. The cells were harvested, suspended in a lysis buffer, and disrupted by sonication. After DNase I treatment, cell lysates were incubated on ice for 3 h for digestion of genomic DNA and centrifuged to remove cell debris. After addition of NaCl and imidazole to make the final concentrations of 1 M and 20 mM, respectively, the cleared cell lysates were adsorbed onto a filter column of nickel-charged nitrilotriacetic acid (NTA) agarose resin (Qiagen). After washing the protein-bound Ni-NTA column with a washing buffer (20 mM imidazole in 50 mM K phosphate buffer containing 500 mM NaCl), the column-bound His-tagged TFs were eluted with an elution buffer (washing buffer containing 250 mM imidazole). The purity of each peak was checked by SDS-PAGE. Peak fractions containing TFs without contaminant proteins were pooled and dialyzed against the storage buffer (20 mM Tris-HCl [pH 7.6] at 4°C, 5 mM Mg acetate, 100 mM NaCl, 0.1 mM EDTA, 1 mM dithiothreitol [DTT], 50% glycerol). The purity of TFs used in this study was more than 90%, as checked by staining of SDS-PAGE gels. PS-TF screening was repeated six times using two or three different batches (see details in the text).

### Preparation of DNA probes.

FITC-labeled DNA probes for PS-TF screening were prepared by PCR amplification of the *rsd* and *rmf* promoter regions indicated in Fig. 1A and B, respectively, using the primers listed in Table S2 in the supplemental material and E. coli K-12 W3110 DNA as the template (for the probe sequences, see Fig. S1). As a reference probe, a 193-bp-long probe was prepared, which corresponded to an open reading frame sequence of the *rtcA* gene encoding RNA 3′-terminal phosphate cyclase.

10.1128/mSystems.00057-18.3 TABLE S2 Primers used in this study. Download Table S2, PDF file, 0.1 MB. Copyright © 2018 Yoshida et al. 2018 Yoshida et al. This content is distributed under the terms of the Creative Commons Attribution 4.0 International license.

10.1128/mSystems.00057-18.4 TABLE S3 Intracellular concentration of TFs. The intracellular concentration of TFs was determined by the quantitative immunoblot method as described by Ishihama et al. (56). The TF concentration was calculated as the relative value to that of RNA polymerase RpoA subunit and is represented as the number of molecules per genome equivalent of DNA. Download Table S3, PDF file, 0.1 MB. Copyright © 2018 Yoshida et al. 2018 Yoshida et al. This content is distributed under the terms of the Creative Commons Attribution 4.0 International license.

10.1128/mSystems.00057-18.5 TABLE S4 (A) Regulatory targets (ArcA plus AcP). (B) Regulatory targets (McbR). (C) Regulatory targets (RcdA). (D) Regulatory targets (SdiA plus HSL analogs). (E) Regulatory targets (SlyA). Download Table S4, PDF file, 0.1 MB. Copyright © 2018 Yoshida et al. 2018 Yoshida et al. This content is distributed under the terms of the Creative Commons Attribution 4.0 International license.

### PS-TF screening *in vitro*.

Using the collection of more than 200 species of purified TFs, we have developed the promoter-specific transcription factor (PS-TF) screening system (38). This PS-TF system was employed in this study for screening TFs with binding activity to the *rsd* and *rmf* promoters. In brief, three DNA probes were constructed by PCR amplification. These DNA probes were mixed with each of 199 species of purified TFs, and after incubation at 37°C for 20 min were directly subjected to PAGE for detection of DNA-protein complexes under the standard conditions (38).

### qRT-PCR.

Total RNA was prepared from E. coli cells using NucleoSpin RNA Plus (Macherey-Nagel). cDNA samples were synthesized using the PrimeScript RT reagent kit (TaKaRa Bio, Inc.). The primers used in qRT-PCR are shown in Table S2. The PCR assays were carried out in a RotorGene6500HRM (Qiagen) device using SYBR Premix *Ex Taq* 2 (TaKaRa Bio, Inc.). In each experiment, total DNA of E. coli (Affymetrix) was added to the reaction mixture. The number of PCR cycles was determined in order to obtain DNA within the linear amplification range from the amplification curve. The copy numbers of samples were obtained after quantitative amplification of the target gene. Threshold cycle (*C_T_*) values of sample DNAs were normalized to the reference *C_T_* values obtained using the known amount of E. coli DNA (91).

### Western blot analysis.

Cells were treated with lysozyme, and whole-cell extracts were prepared by sonication. Total cell proteins were fractionated by Tricine-SDS-PAGE on 15% gels (92, 93) and transferred onto polyvinylidene difluoride (PVDF) membranes (Immobilon-FL transfer membrane; Millipore). Rsd and RMF proteins on membranes were detected with rabbit polyclonal antibodies against Rsd and RMF, respectively (56). The intensity of immunostained bands was measured with ECF substrate (GE Healthcare), using a Typhoon FLA 9000 imager (GE Healthcare) or ImageQuant LAS 4000 (GE Healthcare).

### Detection of Rsd: affinity isolation of RpoD-Rsd complexes.

The expression vector of His-tagged RpoD (pASKA-rpoD; obtained from the NBRP E. coli stock center, National Institute of Genetics, Mishima, Japan) was transformed into test E. coli strains. Cells were inoculated for 9 h in LB medium with 30 μg/ml of chloramphenicol and 50 μM IPTG at 37°C under aeration with constant shaking at 150 rpm. Cells were harvested by centrifugation and lysed in lysis buffer (50 mM Tris-HCl [pH 8.0], 150 mM NaCl, 10% [vol/vol] glycerol). A portion of lysed solution was kept for detection of both Rsd and His-tagged RpoD in total cell extracts. The supernatant was applied to an Ni-NTA agarose column (Qiagen). After the column was washed with 10 column volumes of the lysis buffer, the bound proteins were eluted with lysis buffer containing 200 mM imidazole. Mixtures of 20 μg total cell extracts and 2 μg each of purified Rsd and His-tagged RpoD proteins were subjected to 12% SDS-PAGE. For detection of His-tagged RpoD, the gel was stained with Coomassie blue, while for detection of Rsd, the gel was subjected to Western blot analysis (56) using rabbit anti-Rsd antiserum.

### Measurement of 100S ribosome dimers: sucrose gradient centrifugation.

E. coli was grown in medium E containing 2% polypeptone and 0.5% glucose. Cells were harvested at 2.5, 6, 9, or 24 h after inoculation into fresh medium. Cell pellets were suspended in an association buffer (100 mM NH_4_ acetate, 15 mM Mg acetate, 20 mM Tris-HCl [pH 7.6], 6 mM 2-mercaptoethanol) and mixed with an equal volume of glass beads (212 to 300 μm; Sigma). The homogenate was then centrifuged at 15,000 rpm for 10 min at 4°C. The supernatant was layered on top of a 5 to 20% linear sucrose density gradient made in the association buffer and centrifuged in an SW41 Ti rotor (Beckman) at 40,000 rpm for 1.5 h at 4°C. After centrifugation, the absorbance of the sucrose gradient was measured at 260 nm with a UV-1800 spectrophotometer (Shimadzu, Japan). The ratio between 70S and 100S ribosomes was calculated for each peak by using Systat software for peak separation analysis (Systat Software, Inc., Japan).

### gSELEX screening *in vitro*.

The genomic SELEX (gSELEX) screening was carried out as previously described (58, 59). A mixture of DNA fragments of the E. coli K-12 W3110 genome was prepared after sonication of purified genome DNA and cloned into multicopy plasmid pBR322. In each gSELEX screening, the DNA mixture was regenerated by PCR. For gSELEX screening, 5 pmol of the mixture of DNA fragments and 10 pmol purified His-tagged TF were mixed in a binding buffer (10 mM Tris-HCl [pH 7.8] at 4°C, 3 mM magnesium acetate, 150 mM NaCl, and 1.25 mg/ml bovine serum albumin). The sequences of DNA fragments obtained by the genomic SELEX screening were identified by a SELEX-chip method as described previously (58, 60).
