# Supplementary material for: Coordinated Hibernation of Transcriptional and Translational Apparatus during Growth Transition of Escherichia coli to Stationary Phase
Source: mSystems. 2018 Sep 11;3(5):e00057-18. doi: 10.1128/mSystems.00057-18 (PMC6134199; doi:10.1128/mSystems.00057-18)
Supplement: TABLE S1 [file sys004182257st1.pdf]

[A] Characterized TF

	TF	Y-name	Alternative names	Family	Regulatory function
1	AcrR	YbaH		TetR	Acriflavine resistance regulator
2	AdiY			AraC	Arginine decarboxylase regulator
3	AgaR	YhaW		DeoR	N-Acetylgalactosamine repressor
4	AllR	YbbU	GlxA3	IclR	Allantoin repressor
5	AppY			AraC	Acid phosphatase activator
6	ArcA		CpxC,Dye,FexA,Msp,Seg,SfrA	OmpR	Anoxic redox regulator
7	ArgR		XerA	ArgR	Arginine repressor
8	ArsR		ArsE	ArsR	Arsenate inducibility regulator
9	AscG		Sac	LacI	Arbutin-salicin-cellibiose regulator
10	AsnC			AsnC	Asparagine regulator
11	AtoC			NtrC	Acetoacetate regulator
12	BaeR			OmpR	Bacterial adaptive response regulator
13	BasR		PmrA	OmpR	Bacterial adaptive response regulator
14	BetI			TetR	Betaine inhibitor
15	BirA		BioR,DhbB	BirA	Repressor of biotin operon
16	CadC			CadC	Activator of cadverine synthesis
17	CbpA			CbpA	Curved DNA-binding protein A
18	CitB		CriR,DpiA,MpdA	CitB	Activator of anaerobic citrate catabolism
19	CpxR	YiiA	XpxR	OmpR	Regulator of conjugatge pilus expression
20	Cra		FruC,FruR,Shl	LacI	Catabolite repressor activator
21	CreB	YjjE		OmpR	Carbon source response regulator
22	Crp		Cap,Csm	Crp	cAMP receptor protein
23	CsgD		AgfG,CsgG	LuxR	Regulator of curli-spefic genes
24	CsiR	YgaE	GabC	GntR	Carbon starvation induced regulator
25	CueR	YbbI		MerR	Cu efflux regulator
26	CusR	YlcA	SilR	OmpR	Cu sensing regulator
27	CynR			LysR	Cyanate regulator
28	CytR			LacI	Cytidine regulator
29	Dan	YgiP	TtdR	LysR	DNA-bindng protein under anaerobic conditions
30	DcuR	YjdG		CitB	Regulator of C4-dicarboxylate metabolism
31	DeoR		NucR,NupG,Tsx	DeoR	Regulator of cysteine detoxification
32	DeoT	YciT		DeoR	DeoR-type regulator
33	DhaR	YcgU		NtrC	Dihydroxyacetone regulator
34	DicA		FtsT	Xre	Division control A
35	InfB*			UN	[IF-2 (translation initiatiohn factor IF2)]
36	DicC		FtsT	DicC	Division control C
37	FadR		Dec,OleR,ThdB	GntR	Fatty acid degradation regulator
38	DmlR	YeaT		LysR	D-Malate degradation regulator
39	Dps		PexB,Vtm	Dps	DNA protection during starvation
40	DsdC			LysR	D-Serine deaminase regulator
41	EbgR			LacI	Evolved beta-galactosidase repressor
42	EmrR		MprA	MarR	*E. coli* multidrug resistance regulator
43	EnvR	YhdK	AcrS	TetR	Envelope regulator
44	EvgA			LuxR	*E coli* homolog of Bordetela *bvgS*
45	ExuR			GntR	Exuronate repressor
46	FhlA			NtrC	Formate hydrogen lyase activator
47	Fis		Nbp	Fis	Factor for inversion stimulation
48	FrlR	YfhR		GntR	Fructolysine regulator
49	FrvR	YiiH		AraC	Fructose-like transport regulator
50	FucR			DeoR	Fucose regulator
51	Fur			Fur	Ferric uptake regulator
52	GalR			LacI	Galactose repressor
53	GatR1		GatR (full-size GatR)	DeoR	Galactitol regulator
54	GatR2		GatR (N-terminal fragment-2)	DeoR	Galactitol regulator
55	GlpR			DeoR	Glyceroll-3-phosphate repressor
56	QseF	YfhA	GlrR	NtrC	Quorum-sensing regulator F
57	GusR		UidR	TetR	Glucuronides repressor
58	HcaR	YfhT	PhdR	LysR	Hydrocinnamic acid regulator
59	HdfR	YifA,YifD	PssR	LysR	Hns-dependent flhDC regulator
60	HexR	YebK		RpiR	Hexose regulator
61	Hha			H-NS	High hemolysin activity
62	HigA	YgjM		T-AT	Antitoxin of HigB toxin
63	HipB			T-AT	Antitoxin of HipA toxin
64	Hnr*	YchL	RssB,SprE	UN	[Regulator of RpoS]
65	Hns		BglY,FimG,OzmZ,PilG,TopS,VirR	H-NS	Histone-like nucleoid structuring protein
66	HupA		HU-α,HU-2	HU	Histone-like protein HU-A
67	HupB		DepA,DpeA,HU-β,HU-1	HU	Histone-like protein HU-B
68	ArgP		Can,CanR,IciA	LysR	Arginine protein
69	IclR			IclR	Isocitrate lyase regulator
70	IdnR	YjgS	GntH,GntS	LacI	L-Idonate regulator
71	IhfA		Hid,HimA,IHF-α	HU	Integration host factor A
72	IhfB		FimD,HIP,IHF-β	HU	Integration host factor B
73	IlvY			LysR	Isoleucine-valine regulator
74	KdpE			OmpR	K+-dependent ATPase regulator
75	LacI			LacI	Lactose inhibitor
76	LctR		LldR,Lct	GntR	Lactate regulator
77	LeuO			LysR	Lactate regulator
78	LrhA		GenR,HexA	LysR	LysR homologue A
79	Lrp		AlsB,LblA,LivR,LstR,Mbf,OppI,RblA	AsnC	Leucine-responsive regulatory protein
80	LysR			LysR	Lysine regulator
81	MalI			LacI	Maltose repressor
82	MalP		MalA,Blu	UN	[Maltodoxtrin phosphorylase]
83	MarR		CfxB,InaR,SoxQ	MarR	Multiple antibiotic resistance B
84	McbR	YncC		GntR	MqsR-controlled colanic acid and biofilm regulator
85	Mlc		DgsA	NagC	Regulator to make large conolies
86	MlrA	YehV		MerR	MerR-like regulator of curli production
87	NadR		NadI,PnuA,NMN adenyltransferase	Trigger	Regulator of NAD synthesis
88	MntR	YbiQ		DtxR	Mn2+ transport regulator
89	ModE		ChlD, ModR	ModE	Molybdenum regulator
90	NagC		NagR	NagC	N-Acetylglucosamine regulator
91	NanR	YhcK		GntR	N-Acetyl-neuraminic acid regulator
92	NarL		FrdR,NarR	LuxR	Nitrate/nitrite response regulator NarL
93	NarP			LuxR	Nitrate/nitrite response regulator NarP
94	NemR	YdhM		TetR	N-Ethylmaleimide reductase repressor
95	NhaR	YaaB	AntO	LysR	Na+/H+ antiporter regulator
96	NorR	YgaA		NtrC	NO reduction and detoxification regulator
97	NtrC		GlnG,GlnT	NtrC	Nitrogen regulation C
98	OmpR		Cry,Kmt,OmpB	OmpR	Outer membrane protein regulator
99	OraA*		RecX	UN	[RecA inhibitor]
100	OxyR		MomR,Mor	LysR	Oxidative stress regulator
101	PaaX	YdbY		GntR	Phenylacetic acid regulator
102	PdhR		AceC,GenA	GntR	Pyruvate dehydrogenase complex regulator
103	PepA		CarP,XerB,Aminopeptidase A/I	Trigger	Peptidase regulator
104	PerR			LysR	Peroxide response regulator
105	PhnF			GntR	Phosphonate regulator F
106	PhoB		PhoRc,PhoT	OmpR	Phosphate regulator B
107	CheB*			UN	[Chemotaxis regulator-Glu methyltransferase]
108	PrpR	YahP		NtrC	Propionate regulator
109	PspF	YcjB		NtrC	Phage shock protein F
110	PutA		PoaA	Trigger	Proline utilization regulator
111	PuuR	YcjC		Xre	Putrescine utilization and transport regulator
112	QseA	YhcS	AaeR	LysR	Quorum-sensing regulator A
113	QseB	YgiX	PreA,XgiX	OmpR	Quorum-sensing regulator B
114	QseD	YjiE	HypT	LysR	Quorum-sensing regulator D
115	RbsR			LacI	Ribose repressor
116	RcsA		CpsR	LuxR	Regulator capsule synthesis A
117	RcsB		ViaA	LuxR	Regulator capsule synthesis B
118	SfsB		Nlp	SfsA	Sugar fermentation stimulation regulator B
119	RhaS		RhaC2	AraC	Rhamnose regulator
120	Rob		CbpB,RobA	AraC	Right origin binding protein

121	RpiR	YjcY	AlsR	RpiR	Ribosephosphate isomerase regulator
122	RstA		UrpT	OmpR	Phage RS-region encoded TF
123	RtcR	YhgB		NtrC	RNA terminal phosphate cyclase regulator
124	RutR	YcdC		TetR	Pyrimidine utilization regulator
125	SdiA		UvrX	LuxR	Suppressor of the cell division inhibitor
126	SfsA		Sfs,Sfs1	SfsA	Sugar fermentation stimulation regulator A
127	SlyA			MarR	Hemolytic protein in *Salmonella*
128	SoxR		MarC	MerR	Superoxide response protein R
129	SoxS			AraC	Superoxide response protein S
130	SrlR		GutR	DeoR	Sorbitol repressor
131	StpA		Him3,HnsB,Rsv	H-NS	Suppressor of *td* phenotype A
132	TorR			OmpR	Regulator of TMAO reduction
133	TreR			LacI	Trehalose repressor
134	SlmA	YicB	Ttk	TetR	Synthetically lethal with a defective Min system
135	TyrR			NtrC	Tyrosine repressor
136	UhpA			LuxR	Regulator of uptake of hexose phosphates
137	UlaR	YjfQ		DeoR	Utilization of L-ascorbate regulator
138	UspA			UN	[Universal stress response regulator A]
139	UspC			UN	[Universal stress response regulator B]
140	UvrY	YecB		LuxR	Regulator of repair from UV damage
141	UxuR			GntR	Hexuronate regulator
142	ZntR	YhdM		MerR	Zinc transporter regulator
143	ZraR		HydG	NtrC	Zinc resistance-associated regulator
144	Zur	YjbK		Fur	Zinc uptake regulator
Total, 144 species					

[B] Uncharacterized TFs

	TF	Y-name	Alternative names	Family	Regulatory function
1	YafC			LysR	
2	XynR	YagI		IclR	Regulator of xylonate catabolism
3	YahA			UN	[c-di-GMP phosphodiesterase]
4	YahB			LysR	
5	YahD			UN	[Ankyin repeat protein]
6	DecR	YbaO		AsnC	Regulator of cysteine detoxification
7	YbbO			UN	[NAPD-dependent aldehyde reductase]
8	YbdO			LysR	
9	YbeF			LysR	
10	CecR	YbiH		TetR	Regulator of cefoperazone-chloramphenicol sensitivity
11	RcdA	YbjK		TetR	Regulator of *csgD*
12	YcaN			LysR	
13	ComR	YcfQ		TetR	Copper permeability regulator
14	PgrR	YcjZ		LysR	Regulator of peptidoglycan recycling
15	YdcI			LysR	
16	SutR	YdcN		Xre	Regulator of sulfur utilization
17	YdfH			GntR	
18	YdhB			LysR	
19	NimR	YeaM		AraC	Regulator of 2-imidazole sensitivity
20	HprR	YedW		OmpR	Hydrogen peroxide response regulator
21	YegW			GntR	
22	BtsR	YehT		LytTR	Brenztraubensaure
23	YeiE			LysR	
24	RhmR	YfaX		IclR	*E. coli* PMV-1 regulator
25	YfeR			LysR	
26	YfhH			RpiR	
27	YfiE			LysR	
28	YgaV			ArsR	
29	YgeK			LuxR	
30	YgfB			UN	[UPF0149 family protein]
31	YgfI			LysR	
32	YhaJ			LysR	
33	YhjC			LysR	
34	YiaG			Xre	
35	YiaJ			IclR	
36	YiaU			LysR	
37	YidL			AraC	
38	YidP			GntR	
39	YidZ			LysR	
40	YieP			GntR	
41	YihW			DeoR	
42	YijO			AraC	
43	YjdC			TetR	
44	BdcR	YjgJ		TetR	Biofilm dispersal via c-di-GMP
45	YjiM			UN	[2-Hydroxyglutaryl-CoA dehdratase]
46	YjiR			Trigger	
47	YkgA			AraC	
48	RclR	YkgD		AraC	Reactive chloride resistance regulator
49	YneJ			LysR	
50	YnfL			LysR	
51	YpdB			LytTR	
52	YqhC			AraC	
53	YphH			NagC	
54	YdcR			Trigger	
55	YciT	DeoT		DeoR	DeoR-type regulator
Total, 55 species					
