# Supplementary material for: Coordinated Hibernation of Transcriptional and Translational Apparatus during Growth Transition of Escherichia coli to Stationary Phase
Source: mSystems. 2018 Sep 11;3(5):e00057-18. doi: 10.1128/mSystems.00057-18 (PMC6134199; doi:10.1128/mSystems.00057-18)
Supplement: FIG S1 [file sys004182257sf1.pdf]

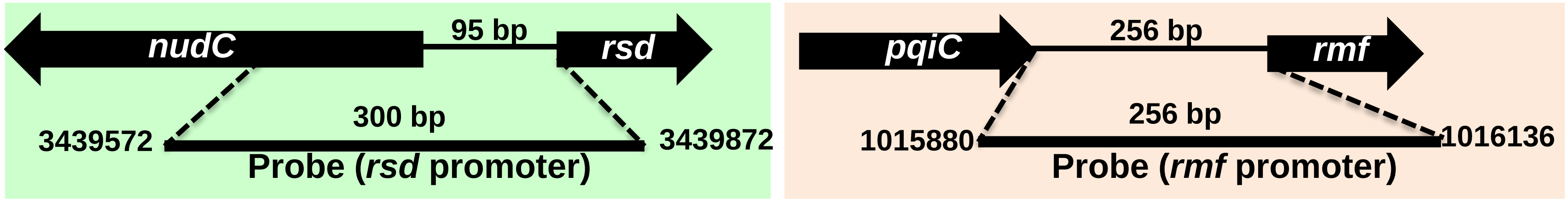

(A) Probe sequence used as the *rsd* promotor (300 bp; genome 3439572-3439872)

**CGTACCGACC CCATATCGTG ACGCCGCTGC TGTTGTACTA ACCAAACAGG TTCCCCCTGC CATTCGCCGA
TTTGTAGTGC GCGCTGACCC ACAAGATCGA AATTTGCCGC TTCGCCATAT GGCAATTCTC CCTTCGGCAA
CCATAATTTT TGTTCATGGC TGACGACCCA CCAGCCGTGA TCTAATTTTT CAATTATACG ATCCATAGCT
CTTGCACTAC CTTTGCATCA CTGGCATGTT TAACATGGTT TTTACATTTC TCACTGAGCA GTTTTTGAAT
ACAAACTTGC GGAGTCAATC**

(B) Probe sequence used as the *rmf* promotor (256 bp; genome 1015880-1016136)

**ATTATAAAGA TTTGTAAATA TAACCGTCTC CGGTATGTTG CCTGAGGCGG TTTTTTTGTC TCTAACGTGC
GGAAAAATTT GTTCCTCTTC ACATTTTTTG TACAACCGAC ATGCCCGTGT AGCTCACAAA TATGACAGTG
GCGTGAATTT TGCGCATTGA CGGCAGTTAT GATTCGCGGT ATTGCTTAAC TGTGATTGCA CATTTAGTAA
TCACTGTTTT CTTTTCCACC AGAAACCAGT ATGAGGGAAA CGAGGC**

(C) Probe sequence used as a reference (193 bp; genome, 3554400-3554600)

**TGGCGTAACA GCGTGGTTTG CTGATGAATT CCTATTTTCG CCAGCAGCGG CTCCAGCACC CGGCGGATAA
AATCCGCAGG CGGCGCCGAC GGGTTATCGG TGCCGCCGCT CACTTCAACA CGCGAAGGTC CATCGGCAAA
CCACAGCGCG GGCAGCACCG TTTGCAGCAC CAGCGTACAA CTTCCGGCGC TAC**

Yoshida et al. (Fig. S1)
