# Supplementary material for: Coordinated Hibernation of Transcriptional and Translational Apparatus during Growth Transition of Escherichia coli to Stationary Phase
Source: mSystems. 2018 Sep 11;3(5):e00057-18. doi: 10.1128/mSystems.00057-18 (PMC6134199; doi:10.1128/mSystems.00057-18)
Supplement: TABLE S2 [file sys004182257st2.pdf]

Supplemental Table S2

Target gene	Primer sequence	
	**Forward**	**Reverse**
rsd	**GCATCTGCTCGTGGCTTAC**	**GCTCTGACAAAAATCATCAAGGGC**
rmf	**GGAACGGGCACATCAACG**	**CCCAGCCATTGTGACCTTTG**
arcA	**AGGCGAATGTTGCGTTGATG**	**GGGTTGAACGGTTTGGTGATG**
mcbR	**CGTCTGGTTTCGGTGAATGC**	**GTAGCGGATCCGATTGATTTCG**
rcdA	**GGAGGCGGTGAAACTTTACG**	**ACGCCTCCAGTAACAACTCATC**
sdiA	**ATGGAGACCGCAGAAGAGG**	**GCCACTTTAGGTCGAGTGAATG**
slyA	**ATGGCGTGCTCTGATAGACC**	**TTTGCGACTGGTCTGGAGG**
