# Supplementary material for: Coordinated Hibernation of Transcriptional and Translational Apparatus during Growth Transition of Escherichia coli to Stationary Phase
Source: mSystems. 2018 Sep 11;3(5):e00057-18. doi: 10.1128/mSystems.00057-18 (PMC6134199; doi:10.1128/mSystems.00057-18)
Supplement: TABLE S3 [file sys004182257st3.pdf]

Supplemental Table S3

TF	No. molecules/cell	
	Exponential phase	Stationary phase
ArcA	90~100	210~240
McbR	40~50	40~50
RcdA	230~250	280~300
SdiA	720~750	840~860
SlyA	250~280	300~330
