# Supplementary material for: Coordinated Hibernation of Transcriptional and Translational Apparatus during Growth Transition of Escherichia coli to Stationary Phase
Source: mSystems. 2018 Sep 11;3(5):e00057-18. doi: 10.1128/mSystems.00057-18 (PMC6134199; doi:10.1128/mSystems.00057-18)
Supplement: TABLE S4 [file sys004182257st4.pdf]

Position	Function	Left gene	D		D	Righ Gene	Function
41946	carnitine BCCT transporter	caiT	<		>	fixA	flavoprotein for anaerobic carnitine reduction
121966	Phe/Tyr/Trp APC transporter	aroP	<		>	pdhR	pyruvate dehydrogenase complex regulator
141230	glucose dehydrogenase	gcd	<		>	hpt	hypoxanthine-Gua phosphoribosyltransferase
243432	acyl-CoA dehydrogenase	fadE	<		>	lpcA	D-sedoheptulose 7-phosphate isomerase
328672	betaine operon transcriptional repressor	betI	<		>	betT	choline BCCT transporter
398634	inner membrane protein	yaiY	<		>	yaiZ	inner membrane protein
450964	cytochrome *bo* terminal oxidase	cyoA	<		>	ampG	muropeptide MFS transporter
478570	inner membrane protein	ylaC	<		<	maa	maltose acetyltransferase
629042	thioesterase for enterobactin biosynthesis	entH	>		>	cstA	peptide transporter
651244	citrate lyase synthetase	citC	<		>	dpiB	DpiBA TCS sensory histidine kinase
655330	DcuC dicarboxylate transporter	dcuC	<		>	pagP	lipid palmitoyltransferase
728256	K+ ion accessory transporter	kdpF	<		>	ybfA	predicted protein
741852	peptide POT Transporter	dtpD	<		>	ybgI	metal-binding hydrolase-oxidase
754030	citrate synthase monomer	gltA	<		>	sdhC	succinate dehydrogenase
770038	alpha-mannosidase	mngB	>		>	cydA	cytochrome *bd* terminal oxidase
840966	catecholate siderophore receptor	fiu	<		<	mcbA	protein involved in colanic acid production
847534	glutamine transporter	glnH	<		<	dps	stationary phase nucleoid iron-binding protein
953940	formate FNT transporter	focA	<		<	ycaO	conserved protein
996834	NAD(P)H-dependent FMN reductase	ssuE	<		>	ycbQ	fimbrial-like adhesin protein
1073354	pyrimidine oxygenase	rutA	<		>	rutR	pyrimidine/purine operon regulator
1084156	heme-containing peroxidase/deferrochelatase	efeB	>		>	phoH	ATP-binding protein
1165242	conserved protein	ycfP	>		>	ndh	NADH:ubiquinone oxidoreductase II
1194272	23S rRNA pseudouridine 2457 synthase	rluE	<		>	icd	isocitrate dehydrogenase
1236756	conserved protein	ycgB	<		>	dadA	D-amino acid dehydrogenase
1359040	glutamate-putrescine ligase	puuA	<		>	puuD	glutamyl-aminobutyrate hydrolase
1402642	*p*-aminobenzoyl-glutamate hydrolase	abgA	<		>	abgR	*p*-aminobenzoyl glutamate operon regulator
1445370	PEA operon transcriptional activator	feaR	<		>	feaB	phenylacetaldehyde dehydrogenase
1451850	oxepin-CoA hydrolase/CoA semialdehyde dehydrogenase	paaZ	<		>	paaA	ring 1,2-phenylacetyl-CoA epoxidase
1486148	conserved protein	ydcF	>		>	aldA	aldehyde dehydrogenase A
1493146	stress-response transcriptional regulator	ydcI	<		>	ydcJ	conserved protein
1561234	D-Ala-D-Ala dipeptidase	ddpX	<		<	dosP	c-di-GMP phosphodiesterase
1684632	fumarase C monomer	fumC	<		<	fumA	fumarase A monomer
1830072	acetylornithine delta-aminotransferase	astC	<		>	xthA	exonuclease III
1887768	acyl-CoA synthetase	fadD	<		<	yeaY	predicted lipoprotein
1977670	IS1 repressor	insA-5	<		>	uspC	universal stress protein
2102542	lipopolysaccharide biosynthesis protein	wbbK	<		<	wbbJ	predicted acyl transferase
2190388	fimbrial-like adhesin protein	yehD	<		<	yehE	predicted protein
2276340	predicted protein	yejG	<		<	bcr	multidrug MFS transporter
2304934	malate:quinone oxidoreductase	mqo	<		<	yojI	multidrug ABC superfamily transporter
2311260	antisense regulator sRNA for *ompF* synthesis	micF	>		>	rcsD	RcsD phosphotransferase
2384870	predicted peptidase	yfbL	>		>	yfbM	predicted protein
2403250	NADH:ubiquinone oxidoreductase	nuoA	<		<	lrhA	type-1 bimbriae operon regulator
2425944	Lys/Arg/ornithine transporter	argT	<		<	ubiX	flavin prenyltransferase
2576570	NADP-dependent malic enzyme	maeB	<		>	talA	transaldolase A
2643032	nucleoside diphosphate kinase	ndk	<		<	pbpC	peptidoglycan glycosyltransferase
2667070	HcaR transcriptional dual regulator	hcaR	<		>	hcaE	3-phenylpropionate dioxygenase
2723936	alpha-ketoglutarate MFS transporter	kgtP	<		<	rrfG	*rrfG* 5S ribosomal RNA
2786834	conserved protein	ygaQ	>		>	csiD	carbon starvation-induced protein
2983750	predicted acyltransferase	yqeF	<		>	yqeG	hydroxy/aromatic amino acid STP transporter
3126130	glycolate oxidase	glcD	<		>	glcC	glycolate operon regulator
3217432	aerotaxis sensor receptor flavoprotein	aer	<		>	ygjG	putrescine aminotransferase
3237758	membrane-bound redox modulator	alx	>		>	sstT	serine/threonine:Na+ symporter
3382536	malate dehydrogenase	mdh	<		>	argR	Arg/His operon regulator
3479338	K+-effulux ancillary protein	kefG	<		>	yheS	ABC superfamily transporter
3537960	transcriptional accessory protein	yhgF	>		>	feoA	ferrous iron transport
3590444	glycerol-3-phosphate transporter	ugpB	<		<	livF	branched chain amino acid ABC transporter
3656246	acid-resistance membrane protein	hdeD	>		>	gadE	glutamic acid operon transcriptional activator
3664032	GadX transcriptional dual regulator	gadX	<		<	gadA	glutamate decarboxylase A
3710232	oxalatae-formate MFS antiporter	yhjX	<		<	yhjY	putative lipase
3775254	conserved protein	yibL	>		>	lldP	dilactate/glycolate:H+ symporter
3921034	ATP-proton motive force interconversion	atpI	<		<	rsmG	16S rRNA G527 methyltransferase
4002730	conserved protein	yigI	<		>	pldA	phospholipase A1
4029138	3-hydroxybutyryl-CoA epimerase	fadB	<		>	pepQ	proline dipeptidase
4158830	pyridine nucleotide transhydrogenase	sthA	<		>	fabR	fatty acid biosynthesis operon regulator
4213436	homoserine O-succinyltransferase	metA	>		>	aceB	malate synthase A
4285548	acetyl-CoA synthetase	acs	<		>	nrfA	cytochrome C552
4323268	phosphonate ABC transporter	phnC	<		<	yjdN	conserved protein
4360056	CadC transcriptional activator	cadC	<		<	pheU	tRNA-Phe
4364850	dicarboxylate transporter	dcuA	<		<	aspA	aspartate ammonia-lyase
		Total no. binding sites	69				
		Total no. regulatory targets	102				

Position	Function	Gene	D	No	AI2	D	Gene	Function
34072	carbamoyl-phosphate synthase	***carB***	>			>	***caiF***	carnitine operon regulator
264544	CP4-6 prophage protein	***yafX***	<			<	***ykfF***	CP4-6 prophage protein
313130	*rpmJ* (L36) paralog	***ykgM***	<			>	***insE***	IS3 element protein *InsE*
354170	propionyl-CoA synthetase	***prpE***	>			>	***codB***	cytosine transporter
407866	conserved protein	***yaiE***	>			<	***rdgC***	nucleoid-binding RecA regulator
527330	predicted protein	***ybbC***	>			<	***ybbB***	tRNA 2-selenouridine synthase
558062	fimbrial-like adhesin protein	***sfmA***	>			>	***sfmC***	pilin chaperone
636838	DNA-binding transcriptional regulator	***ybdO***	<			<	***dsbG***	periplasmic disulfide isomerase
660856	DNA-binding transcriptional regulator	***ybeF***	<			<	***lipB***	lipoyl-protein ligase
688452	IS5 transposase and trans-activator	***insH***	<			<	***lnt***	apolipoprotein N-acyltransferase
707052	glutamyl-tRNA synthetase	***glnS***	>			>	***ybfM***	outer membrane porin
728772	predicted protein	***ybfA***	>			>	***rhsC***	*rhsC* element core protein RshC
1084156	conserved protein	***efeB***	>			>	***phoH***	nucleoside triphosphate hydrolase
1200736	e14 prophage excisionase	***xisE***	<			>	***ymfI***	e14 prophage protein
1314070	predicted protein	***yciG***	<			<	***trpA***	tryptophan synthase
1332842	Cys synthesis operon regulator	***cysB***	>			>	***ymiA***	hypothetical protein
1342734	hypothetical protein	***yciZ***	<			<	***gmr***	modulator of RNase II stability
1926870	protease II	***ptrB***	<			<	***yebE***	conserved protein
2020940	flagellar biosynthesis protein	***fliQ***	>			>	***fliR***	flagellar export pore protein
2032840	ncRNA	***rseX***	>			>	***hchA***	Hsp31 molecular chaperone
2065942	IS5 transposase and trans-activator	***insH***	<			<	***insD***	KpLE2 IS2 insertion element transposase
2105234	O-antigen polymerase	***rfc***	<			<	***glf***	UDP-galactopyranose mutase
2175434	D-tagatose 1,6-bisphosphate aldolase 2	***gatY***	<			<	***fbaB***	fructose-bisphosphate aldolase class I
2331448	predicted protein	***yfaQ***	<			<	***yfaT***	predicted protein
2594748	conserved protein	***ypfJ***	<			<	***purC***	ribosylimidazole-succinocarboxamide synthase
2773032	CP4-57 prophage inner membrane protein	***yfjW***	>			>	***yfjX***	CP4-57 prophage antirestriction protein
2802570	ribonucleoside-diphosphate reductase 2	***nrdF***	>			>	***proV***	glycine betaine transporter subunit
2992436	pathogenesis-related protein	***ygeI***	>			<	***insD***	KpLE2 phage IS2 transposase
3110762	secretion pathway M-type protein	***yghD***	<			<	***yghG***	predicted protein
3132050	nucleoside triphosphate hydrolase domain	***yghS***	<			>	***yghT***	ATP-binding NTP hydrolase
3144758	predicted protein	***yghW***	<			>	***yghZ***	aldo-keto reductase
3517370	shikimate kinase I	***aroK***	<			<	***hofQ***	predicted fimbrial transporter
3537860	radiation-response translation control	***yhgF***	>			>	***feoA***	ferrous iron transporter
3597672	leucine/isoleucine/valine transportert	***livJ***	<			<	***rpoH***	RNA polymerase, sigma 32 (sigma H)
3664032	GAD-depend acid resistance regulator	***gadX***	<			<	***gadA***	glutamate decarboxylase A
3976654	acetamino-dideoxy-galactose transferase	***rffT***	>			>	***wzyE***	ECA polysaccharide chain elongation protein
4148470	inner membrane protein	***yijP***	<			<	***ppc***	phosphoenolpyruvate carboxylase
4151358	phosphoenolpyruvate carboxylase	***ppc***	<			<	***argE***	acetylornithine deacetylase
4166466	Glu tRNA	***gltT***	>			>	***rrlB***	23S ribosomal RNA of rrnB operon
4207870	Glu tRNA	***gltV***	>			>	***rrlE***	23S ribosomal RNA of *rrnE* operon
4259732	predicted protein	***yjbM***	>			>	***dusA***	tRNA-dihydrouridine synthase A
4267172	hypothetical protein	***yjbS***	<			>	***aphA***	acid phosphatase/phosphotransferase
	**Total no. binding sites**			**27**	**26**			
	**Total no. regulatory targets**			**38**	**37**			

Supplemental Table S4C

Position	Function	Gene	D		D	Gene	Function
19830	InsAB transposase	*insAB-1*	<		<	*insB-1*	IS1 protein InsB
384316	conserved protein	*yaiS*	<		>	*tauA*	taurine ABC transporter
582848	DLP12 prophage protein	*tfaX*	>		>	*appY*	DNA binding transcriptional activator
584962	outer membrane protease VII	*ompT*	<		<	*envY*	EnvY transcriptional activator
886556	predicted transporter	*ybjJ*	<		>	*rcdA*	DNA-binding transcriptional regulator
1020144	SOS cell division inhibitor	*sulA*	<		>	*sxy*	transcriptional coactivator for CRP
1102554	transcriptional dual regulator	*csgD*	<		>	*csgB*	curlin, minor subunit precursor
1104940	predicted protein	*ymdA*	>		>	*ymdB*	regulator of RNase III activity
1209454	e14 site-specific DNA recombinase	*pinE*	>		>	*mcrA*	restriction at 5-methylcytosine residues
1214962	blue light-responsive regulator of YcgE	*ycgF*	<		>	*ycgZ*	predicted protein
1321242	*trp* operon leader peptide	*trpL*	<		>	*yciV*	conserved protein
1622770	stress response protein	*ydeI*	<		>	*ydeJ*	conserved protein
1631666	Qin prophage transcriptional regulator	*ydfK*	>		>	*pinQ*	Qin prophage site-specific recombinase
1638666	Qin prophage S lysis protein	*essQ*	<		<	*cspB*	Qin cold shock transcriptional regulator
1669136	MFS transporter	*ynfM*	>		>	*asr*	acid shock protein
1669932	small membrane protein	*ydgU*	>		>	*ydgD*	predicted peptidase
1976556	InsAB' transposase	*insAB-5*	<		<	*insB-5*	IS1 protein
2066688	IS5 transposase and trans-activator	*insH-6*	<		>	*yoeA*	CP4-44 disrupted hemin or colicin receptor
2264250	NAD-dependent dehydrogenase	*yeiP*	>		>	*yeiQ*	predicted NAD-dependentdehydrogenase
2311454	antisense RNA against OmpF	*micF*	>		>	*rcsD*	RcsD phosphotransferase
2439732	β-ketoacyl-[acyl carrier prot] synthase I	*fabB*	<		>	*mnmC*	methylaminomethyl-thioU methyltransferase
2797030	nucleoid-protein with RNA chaperone activity	*stpA*	<		>	*ygaW*	predicted inner membrane protein
2989270	predicted protein	*ygeF*	>		>	*ygeG*	predicted chaperone
3651858	uncharacterized protein (pseudogene)	*yhiS_2*	<		>	*slp*	starvation lipoprotein
3766966	conserved protein	*yibG*	>		>	*yibV*	hypothetical protein
4042234	protein disulfide oxidoreductase	*dsbA*	>		>	*yihF*	putative GTP-binding protein
4328334	conserved protein	*yjcZ*	>		>	*proP*	osmolyte:H+ symporter
4336030	AdiY transcriptional activator	*adiY*	<		<	*adiA*	arginine decarboxylase
4477770	predicted acetyltransferase	*yjgM*	<		>	*yjgN*	conserved inner membrane protein
4502056	IS1 predicted transposase	*insG*	<		>	*yjhB*	MFS transporter
4538758	N-acetylneuraminic acid OM channel	*nanC*	<		>	*fimB*	regulator for *fimA*
		**Total no. binding sites**		**31**			
		**Total no. regulatory targets**		**44**			

Supplemental Table S4D

Position	Function	Left gene	D	N	A	F	K	D	Right gene	Function
11936	predicted protein	*yaaI*	<					>	*dnaK*	chaperone Hsp70, co-chaperone with DnaJ
29360	dihydrodipicolinate reductase	*dapB*	>					>	*carA*	carbamoyl P synthetase, Gln amidotransferase
84246	*leu* operon leader peptide	*leuL*	<					>	*leuO*	DNA-binding transcriptional activator
85462	Leu regulon transcriptional regulator	*leuO*	>					>	*ilvI*	acetolactate synthase III
89272	carbon metabolism transcriptional regulator	*cra*	>					>	*mraZ*	conserved protein
121872	aromatic amino acid transporter	*aroP*	<					>	*pdhR*	pyruvate dehydrogenase complex regulator
312342	*rpmJ* (L36) paralog	*ykgM*	<					>	*insE*	IS3 element protein InsE
317836	predicted protein	*ykgI*	<					<	*ykgC*	oxidoreductase FAD/NAD(P)-binding domain
440564	exonuclease VII small subunit	*xseB*	<					>	*thiI*	sulfurtransferase for thiamine/4-thiouridinesynthesis
454130	PBP regulator lactamase transcription	*bolA*	>					>	*tig*	peptidyl-prolyl cis/trans isomerase (trigger factor)
568022	DLP12 prophage multidrug resistance protein	*emrE*	>					>	*ybcK*	DLP12 prophage recombinase
576232	IS5 transposase and trans-activator	*insH*	<					>	*essD*	DLP12 prophage phage lysis protein
651244	citrate lyase synthetase	*citC*	<					>	*dpiB*	CitBA citrate operon TCS histidine kinase
655330	anaerobic C4-dicarboxylate transport	*dcuC*	<					>	*pagP*	palmitoyl transferase for Lipid A
660672	DNA-binding transcriptional regulator	*ybeF*	<					<	*lipB*	lipoyl-protein ligase
702948	glucosamine-6-phosphate deaminase	*nagB*	<					>	*nagE*	N-acetyl glucosamine specific PTS enzyme II
735540	predicted protein	*ybfC*	>					>	*ybfQ*	predicted transposase
735932	transposase	*ybfQ*	>					>	*ybfD*	conserved protein
770460	alpha-mannosidase	*mngB*	>					>	*cydA*	cytochrome d terminal oxidase, subunit I
832234	conserved protein	*ybiA*	<					>	*dinG*	ATP-dependent DNA helicase
840966	iron outer membrane transporter	*fiu*	<					<	*mcbA*	predicted protein
849470	threonine and homoserine efflux system	*rhtA*	<					>	*ompX*	outer membrane protein
953940	formate transporter	*focA*	<					<	*ycaO*	conserved protein
986332	outer membrane porin 1a	*ompF*	<					<	*asnS*	asparaginyl tRNA synthetase
996834	NAD(P)H-dependent FMN reductase	*ssuE*	<					>	*ycbQ*	fimbrial-like adhesin protein
1064672	predicted protein	*yccE*	>					>	*agp*	glucose-1-phosphatase/inositol phosphatase
1091836	outer membrane protein	*pgaA*	<					>	*ycdT*	predicted diguanylate cyclase
1120372	predicted protein	*bssS*	<					<	*dinI*	DNA damage-inducible protein I
1214838	FAD-binding phosphodiesterase	*ycgF*	<					>	*ycgZ*	predicted protein
1271162	calcium/sodium:proton antiporter	*chaA*	<					>	*chaB*	predicted cation regulator
1314248	predicted protein	*yciG*	<					<	*trpA*	tryptophan synthase
1402642	aminobenzoyl-glutamate utilization protein	*abgA*	<					>	*abgR*	aminoenzoyl-glutamate operon regulator
1431862	Rac prophage site-specific recombinase	*pinR*	<					<	*ynaE*	Rac prophage transcriptional regulator
1544148	predicted protein	*yddJ*	<					<	*yddG*	methyl viologen efflux pump
1570156	glutamate decarboxylase B, PLP-dependent	*gadB*	<					<	*pqqL*	predicted peptidase
1580646	conserved protein	*ydeN*	<					<	*ydeO*	acid response Gad cascade regulator
1585730	fimbrial-like adhesin protein	*ydeQ*	<					<	*ydeR*	fimbrial-like adhesin protein
1621964	conserved protein	*ydeH*	<					<	*ydeI*	conserved protein
1622654	conserved protein	*ydeI*	<					>	*ydeJ*	conserved protein
1631434	Qin prophage acid-response regulator	*ydfK*	>					>	*pinQ*	Qin prophage site-specific recombinase
1653764	conserved inner membrane protein	*ynfA*	<					>	*ynfB*	predicted protein
1702472	predicted oxidoreductase	*ydgJ*	<					>	*blr*	beta-lactam resistance membrane protein
1719066	hemolytic protein-lke transcriptional regulator	*slyA*	<					>	*ydhI*	inner membrane protein
1752832	4Fe-4S ferridoxin-type protein	*ydhY*	<					<	*ydhZ*	predicted protein
1755332	pyruvate kinase I	*pykF*	>					>	*lpp*	murein lipoprotein
1801348	threonyl-tRNA synthetase	*thrS*	<					>	*yniD*	predicted protein
1804342	conserved protein	*ydiY*	<					>	*pfkB*	6-phosphofructokinase II
1811260	predicted protein	*ydjO*	<					<	*cedA*	cell division modulator
1821336	Gln-dependent NAD synthase	*nadE*	>					>	*cho*	endonuclease of nucleotide excision repair
1823762	β-ketoacyl-[acyl carrier protein] synthase I	*spy*	<					<	*astE*	succinylglutamate desuccinylase
1842870	inner membrane protein	*ynjI*	<					<	*topB*	DNA topoisomerase III
1906068	predicted protein	*yobF*	<					<	*yebO*	predicted protein
1944202	RuvABC resolvasome	*ruvA*	<					>	*yebB*	predicted protein
2033552	ncRNA	*rseX*	>					>	*hchA*	Hsp31 molecular chaperone
2051334	adhesin	*yeeJ*	>					>	*shiA*	shikimate transporter
2066456	IS5 transposase and trans-activator	*insH*	<					<	*insD*	KpLE2 IS2 transposase InsAB
2310860	outer membrane porin protein C	*ompC*	<					>	*micF*	ncRNA
2311260	ncRNA	*micF*	>					>	*rcsD*	RcbBC phosphotransfer intermediate protein
2347662	conserved protein	*inaA*	<					<	*glpQ*	glycerophosphodiester phosphodiesterase
2363766	conserved protein	*ais*	<					>	*arnB*	Uri-5' (pentapyranosyl-ulose PPi) aminotransferase
2467134	CPS-53 bactoprenol glucosyl transferase	*yfdH*	>					>	*yfdI*	CPS-53 (KpLE1) prophage IM protein
2475638	D-Ser operon transcriptional regulator	*dsdC*	<					>	*dsdX*	predicted transporter
2491366	formyl-CoA transferase,	*frc*	<					<	*yfdX*	predicted protein
2560064	CPZ-55 prophage protein	*yffN*	>					>	*yffO*	CPZ-55 prophage protein
2561242	CPZ-55 prophage protein	*yffP*	>					>	*yffQ*	CPZ-55 prophage protein
2627762	predicted protein	*yfgG*	>					>	*yfgH*	outer membrane lipoprotein
2714546	pyruvate formate lyase subunit	*yfiD*	<					>	*ung*	uracil-DNA-glycosylase
2729552	16S ribosomal RNA of *rrnG* operon	*rrsG*	<					<	*clpB*	protein disaggregation chaperone
2773032	CP4-57 prophage inner membrane protein	*yfjW*	>					>	*yfjX*	CP4-57 prophage antirestriction protein
2781672	adhesin-like autotransporter	*ypjA*	<					<	*ileY*	Ile tRNA
2784564	Ile tRNA	*ileY*	<					>	*csiD*	predicted protein
2796642	nucleoid-associated H-NS-like regulator	*stpA*	<					>	*ygaW*	inner membrane protein
2802658	ribonucleoside-diphosphate reductase 2	*nrdF*	>					>	*proV*	glycine betaine transporter
2816770	Ser tRNA	*serV*	<					<	*csrA*	carbon source metabolism regulator
2882356	predicted protein	*ygcL*	<					<	*ygcB*	DEAD box family protein
2925954	conserved protein	*ygdH*	>					>	*sdaC*	serine transporter
2931960	L-fuculose-1-phosphate aldolase	*fucA*	<					>	*fucP*	L-fucose transporter
2945334	murein transglycosylase A	*mltA*	<					>	*metZ*	Met tRNA
2985254	predicted transporter	*yqeG*	>					>	*yqeH*	conserved protein
3004034	AI2-induced transcriptional regulator	*ygeV*	<					>	*ygeW*	conserved protein
3077446	mannitol-specific PTS enzyme IIA	*cmtB*	<					<	*tktA*	transketolase 1, thiamin-binding
3117230	inner membrane lipoprotein	*yghJ*	<					<	*glcA*	glycolate transporter
3134540	phosphate transporter	*pitB*	<					<	*gsp*	glutathionylspermidine amidase
3144338	hydrogenase 2	*hybO*	<					<	*yghW*	predicted protein
3170266	inner membrane protein	*ygiZ*	<					>	*mdaB*	NADPH quinone reductase

3204358	anaerobic growth nucleoid protein	***dan***	<					>	***ttdA***	L-tartrate dehydratase
3214730	siderophore interacting protein	***yqjH***	<					>	***yqjI***	transcriptional regulator
3217358	aerotaxis signal transducer sensory	***aer***	<					>	***ygjG***	putrescine:2-oxoglutaric acid aminotransferase
3265162	Thr/Ser regulonl regulator	***tdcA***	<					>	***tdcR***	Thr/Ser regulonl regulator
3279834	N-acetylgalactosamine-specific PTS	***agaV***	>					>	***agaS***	tagatose-6-phosphate ketose/aldose isomerase
3359040	glutamate synthase, 4Fe-4S proteint	***gltD***	>					>	***gltF***	periplasmic protein
3427154	16S ribosomal RNA of *rrnD* operon	***rrsD***	<					>	***yrdA***	conserved protein
3316172	conserved protein	***yhbC***	<					<	***metY***	Met tRNA
3348572	isoprenoid biosynthesis protein	***elbB***	<					>	***ryhA***	ncRNA
3375554	stringent starvation protein A	***sspA***	<					<	***rpsI***	30S ribosomal subunit protein S9
3411666	drug-efflux regulon transcriptional regulator	***envR***	<					>	***acrE***	cytoplasmic membrane lipoprotein
3427154	16S ribosomal RNA of rrnD operon	***rrsD***	<					>	***yrdA***	conserved protein
3530664	inner membrane protein	***yhgE***	<					>	***pck***	phosphoenolpyruvate carboxykinase
3550864	maltodextrin phosphorylase	***malP***	<					>	***malT***	maltotriose-ATP-binding transcription regulator
3581134	conserved protein	***yhhZ***	>					>	***insA***	KpLE2 phage-like element; IS1 repressor InsA
3595724	leucine transporter subunit	***livK***	<					>	***yhhK***	conserved protein
3597838	leucine/isoleucine/valine transporter	***livJ***	<					<	***rpoH***	RNA polymerase sigma 32 (sigma H) factor
3622268	predicted protein	***yhhH***	>					>	***yhhI***	predicted transposase
3632570	predicted protein	***yhiJ***	<					>	***yhiM***	inner membrane protein
3670238	YhjAB TCS response regulator	***yhjB***	<					>	***yhjC***	DNA-binding transcriptional regulator
3706050	dipeptide transporter	***dppA***	<					<	***proK***	Pro tRNA
3728944	D-xylose isomerase	***xylA***	<					>	***xylF***	D-xylose transporter subunit
3802172	UDP-D-galactose:LPS-galactosyltransferase	***rfaB***	<					<	***rfaS***	LP core biosynthesis protein
3802986	UDP-D-galactose:LPS-galactosyltransferase	***rfaI***	<					<	***rfaB***	UDP-D-galactose:LP-1,6-D-galactosyltransferase
3803124	LPS core biosynthesis protein	***rfaS***	<					<	***rfaP***	LP phosphorylates core heptose kinase
3806372	LPS core biosynthesis protein	***rfaQ***	<					>	***waaA***	3-deoxy-D-manno-octulosonic-acid transferase
3834134	predicted transporter	***yicJ***	<					>	***selC***	Sec tRNA
3841848	xanthine/uracil permase	***yicO***	<					>	***ade***	cryptic adenine deaminase
3886640	tryptophanase leader peptide	***tnaC***	>					>	***tnaA***	tryptophanase/L-cysteine desulfhydrase
3904730	*bgl* operon transcriptional antiterminator	***bglG***	<					<	***phoU***	PhoRB two-component regulator
3920768	membrane-bound ATP synthase	***atpI***	<					<	***gidB***	SAM-dependent methyltransferase
3929268	transcriptional regulator: sigma-54 activator	***ravA***	<					>	***kup***	potassium transporter
4044850	predicted endonuclease	***yihG***	<					>	***polA***	DNA polymerase I
4076772	predicted acetyltransferase	***yiiD***	>					>	***yiiE***	DNA-depemdemt transcriptional regulator
4233870	glucosephosphate isomerase	***pgi***	>					>	***yjbE***	predicted protein
4249834	maltose regulon periplasmic protein	***malM***	>					>	***ubiC***	chorismate pyruvate lyase
4267058	hypothetical protein	***yjbS***	<					>	***aphA***	acid phosphatase/phosphotransferase
4292346	heme lyase (NrfEFG)	***nrfG***	>					>	***gltP***	glutamate/aspartate:proton symporter
4324976	conserved protein	***yjdM***	<					>	***yjdA***	nucleoside triphosphate hydrolase kinase
4402644	inner membrane protein	***yjeT***	>					>	***purA***	adenylosuccinate synthetase
4539836	Tyr recombinase/inversion *fimA* on/off regulator	***fimB***	>					>	***fimE***	Tyr recombinase/inversion *fimA* on/off regulator
4554598	predicted protein	***yjiC***	<					>	***yjiD***	DNA replication/recombination/repair protein
4589632	inner membrane protein	***yjiY***	<					>	***tsr***	methyl-accepting chemotaxis Ser sensor
4601072	inner membrane protein	***yjjP***	<					>	***yjjQ***	DNA-binding transcriptional regulator
			**Total no. binding sites**	**48**	**77**	**67**	**57**			
			**Total no. regulatory targets**	**70**	**##**	**97**	**78**			

Supplemental Table S4E

Function	Gene	D		D	Gene	Function
leu operon leader peptide	*leuL*	<		>	*leuO*	*leu* regulon regiator
Asp tRNA	*aspV*	>		>	*yafT*	predicted aminopeptidase
predicted protein	*yaiA*	>		>	*aroM*	conserved protein
predicted protein	*tomB*	<		<	*acrB*	multidrug efflux system protein
IS5 transposase and trans-activator	*insH*	<		>	*essD*	DLP12 prophage phage lysis protein
predicted protein	*ybfA*	>		>	*rhsC*	*rhsC* element core protein RshC
citrate synthase	*gltA*	<		>	*sdhC*	succinate dehydrogenase
alpha-mannosidase	*mngB*	>		>	*cydA*	cytochrome *d* terminal oxidase
periplasmic protein	*tolB*	>		>	*pal*	peptidoglycan-associated OM lipoprotein
Rossmann-fold domain transferase	*ybhK*	<		>	*moaA*	molybdopterin biosynthesis protein A
aquaporin	*aqpZ*	<		>	*ybjD*	nucleoside triphosphate hydrolase
formate transporter	*focA*	<		<	*ycaO*	conserved protein
ferric-rhodotorulic acid OM transporter	*fhuE*	<		>	*hinT*	purine nucleoside phosphoramidase
hemolysin E	*hlyE*	<		>	*umuD*	DNA polymerase V
ABC superfamily MD transporter	*yddA*	<		<	*ydeM*	conserved protein
predicted lipoprotein	*ydeK*	<		<	*lsrK*	autoinducer-2 (AI-2) kinase
predicted protein	*ynfD*	>		>	*ynfE*	oxidoreductase
autogeneous regulation	*slyA*	<		>	*ydhI*	inner membrane protein
predicted transporter	*ydhC*	>		>	*cfa*	cyclopropane fatty acyl phospholipid synthase
threonyl-tRNA synthetase	*thrS*	<		>	*yniD*	predicted protein
predicted protein	*yoaI*	<		>	*yeaL*	conserved inner membrane protein
conserved protein	*yoaB*	>		>	*yoaC*	predicted protein
predicted protein	*yobF*	<		<	*yebO*	predicted protein
flagellar regulon transcriptional regulator	*flhD*	<		<	*insB*	IS1 transposase InsAB'
cell division control transcriptional activator	*sdiA*	<		<	*yecC*	ABC superfamily transporter
IS5 transposase and trans-activator	*insH*	<		<	*insD*	KpLE2 IS2 transposase InsAB'
predicted protein	*yegR*	<		>	*yegS*	phosphatidylglycerol kinase
ncRNA	*micF*	>		>	*rcsD*	RcsBC TCS phosphotransfer intermediate
adhesin	*yfaL*	<		<	*ypaB*	hypothetical protein
outer membrane porin protein	*yfaZ*	<		>	*nudI*	predicted NUDIX hydrolase
CPS-53 (KpLE1) prophage protein	*yfdP*	>		>	*yfdQ*	CPS-53 (KpLE1) prophage protein
Mur-NAC cell wall regulator	*murR*	<		>	*murQ*	predicted PTS component
predicted inner membrane protein	*yfgF*	<		>	*yfgG*	predicted protein
Ile tRNA	*ileY*	<		>	*csiD*	predicted protein
predicted protein	*ygdR*	>		>	*tas*	NADP(H)-dependent oxidoreductase
arabinose transporter	*araE*	<		<	*kduD*	2-deoxy-D-gluconate 3-dehydrogenase
predicted transporter	*yqeG*	>		>	*yqeH*	conserved protein
ABC superfamily transporter	*ygiS*	<		<	*mqsA*	MqsRA T-AT antitoxin regulator
KpLE2 phage IS2 transposase InsAB'	*insD*	>		>	*yqiH*	predicted periplasmic pilin chaperone
predicted glycogen synthesis protein	*glgS*	<		>	*yqiJ*	predicted inner membrane protein
arg operon transcriptional dual regulator	*argR*	>		>	*yhcN*	conserved protein
predicted outer membrane protein	*yhdV*	>		>	*yhdX*	predicted amino-acid transporter
DNA-binding transcriptional dual regulator	*gadX*	<		<	*gadA*	glutamate decarboxylase A
predicted glutathione S-transferase	*yibF*	<		>	*rhsA*	*rhsA* element core protein RshA
bifunctional enzyme/transcriptional regulator	*yifB*	<		>	*ilvL*	*ilvG* operon leader peptide
ilvG operon leader peptide	*ilvL*	>		>	*ilvM*	acetolactate synthase II
predicted endonuclease	*yihG*	<		>	*polA*	DNA polymerase I
predicted acetyltransferase	*yiiD*	>		>	*yiiE*	transcriptional regulator
glyoxylate bypass operon regulator	*iclR*	<		>	*metH*	homoCys-methyltetrahydrofolate transmethylase
biodegradative arginine decarboxylase	*adiA*	<		<	*melR*	DNA-binding transcriptional dual regulator
PAPS-bisphosphate nucleotidase	*cysQ*	>		>	*ytfI*	predicted protein
trehalose transport catabolism regulator	*treR*	<		>	*mgtA*	magnesium transporter
KpLE2 RNAP sigma 19 factor	*fecI*	<		>	*insA*	KpLE2 phage IS1 repressor protein InsA
conserved protein	*yjhX*	<		<	*yjhS*	conserved protein
N-acetylnuraminic acid OM channel protein	*nanC*	<		>	*fimB*	tyrosine recombinase/inversion *fimA* regulator
tyrosine recombinase/*fimA on-off* regulator	*fimB*	>		>	*fimE*	tyrosine recombinase/inversion *fimA* regulator
predicted protein	*yjiC*	<		>	*yjiD*	DNA replication/recombination/repair protein
**Total no. binding sites**		56				
**Total no. regulatory targets**		80				
